# Bactericidal and Cytotoxic Properties of Silver Nanoparticles

**DOI:** 10.3390/ijms20020449

**Published:** 2019-01-21

**Authors:** Chengzhu Liao, Yuchao Li, Sie Chin Tjong

**Affiliations:** 1 Department of Materials Science and Engineering, Southern University of Science and Technology, Shenzhen 518055, China; 2 Department of Materials Science and Engineering, Liaocheng University, Liaocheng 252000, China; liyuchao@lcu.edu.cn; 3 Department of Physics, City University of Hong Kong, Tat Chee Avenue, Kowloon, Hong Kong, China

**Keywords:** silver ion, bacteria, cytotoxicity, cell culture, membrane, reactive oxygen species, polymer nanocomposite, food packaging, wound dressing, administration route

## Abstract

Silver nanoparticles (AgNPs) can be synthesized from a variety of techniques including physical, chemical and biological routes. They have been widely used as nanomaterials for manufacturing cosmetic and healthcare products, antimicrobial textiles, wound dressings, antitumor drug carriers, etc. due to their excellent antimicrobial properties. Accordingly, AgNPs have gained access into our daily life, and the inevitable human exposure to these nanoparticles has raised concerns about their potential hazards to the environment, health, and safety in recent years. From in vitro cell cultivation tests, AgNPs have been reported to be toxic to several human cell lines including human bronchial epithelial cells, human umbilical vein endothelial cells, red blood cells, human peripheral blood mononuclear cells, immortal human keratinocytes, liver cells, etc. AgNPs induce a dose-, size- and time-dependent cytotoxicity, particularly for those with sizes ≤10 nm. Furthermore, AgNPs can cross the brain blood barrier of mice through the circulation system on the basis of in vivo animal tests. AgNPs tend to accumulate in mice organs such as liver, spleen, kidney and brain following intravenous, intraperitoneal, and intratracheal routes of administration. In this respect, AgNPs are considered a double-edged sword that can eliminate microorganisms but induce cytotoxicity in mammalian cells. This article provides a state-of-the-art review on the synthesis of AgNPs, and their applications in antimicrobial textile fabrics, food packaging films, and wound dressings. Particular attention is paid to the bactericidal activity and cytotoxic effect in mammalian cells.

## 1. Introduction

**Figure 1:**
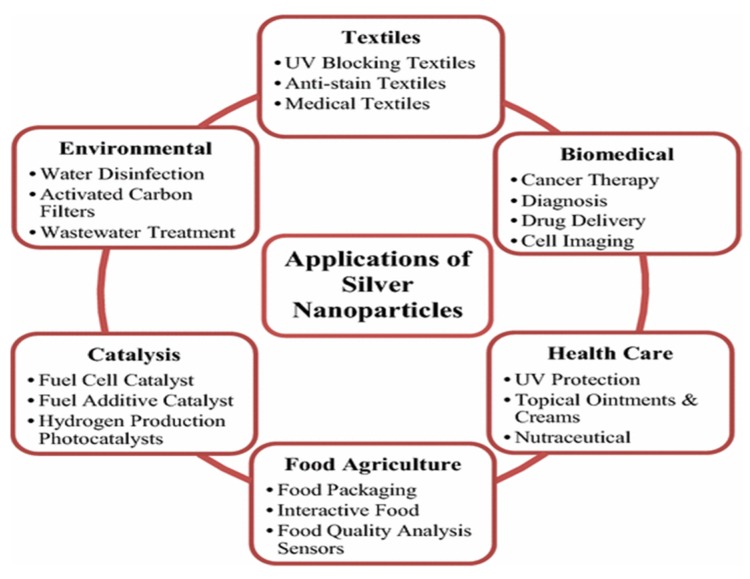
Applications of AgNPs. Reproduced from [22], Springer Open.

Nanotechnology is a multidisciplinary field that brings together researchers in diverse scientific disciplines such as biology, chemistry, material science and physics for developing advanced functional materials at the nanoscale level. The physicochemical and biological properties of nanomaterials differ significantly from the corresponding bulk materials due to their extremely large surface area to volume ratio. Recent advances in nanotechnology allow the synthesis of various types of novel nanomaterials for industrial and biomedical applications [1,2,3,4,5,6,7,8,9,10]. Among these, metal nanoparticles with unique optical properties have gained much attention in the field of nanomedicine, for drug delivery, imaging, and sensing purposes [11,12,13]. In particular, silver nanoparticles (AgNPs) exhibit several attractive properties, including excellent electrical conductivity, chemical stability, antifungal, and bactericidal properties. As such, AgNPs find attractive applications in textiles, healthcare products, cosmetics, cancer therapies, wound dressings, catalysts, food packaging films, water treatments, electronic devices, corneal replacements, etc. (Figure 1) [14,15,16,17,18,19,20,21,22,23,24,25,26,27,28,29]. For example, clothing textiles that are in close contact with human skin create a warm and humid environment for microorganisms. Sweaty fabrics are perfect breeding grounds for bacteria. Thus, antimicrobial fabrics are coated with AgNPs to inhibit bacterial adhesion and growth [30,31]. Biomedical products with AgNPs are typically used to prevent bacterial infections by accelerating wound healing [21,27,28,29], and treating tumor through a rapid breakdown of infected cells [24,25]. AgNPs with doxorubicin and alendronate serve as effective antitumor drug carriers for the HeLa cell line [13].

**Figure 2:**
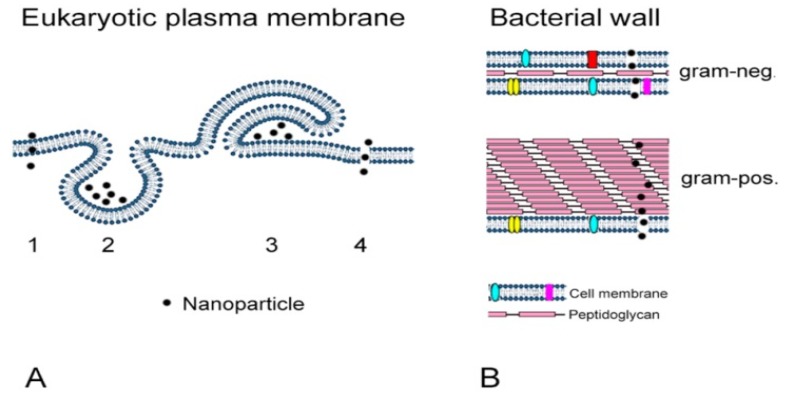
Uptake of AgNPs by mammalian cells (**A**) and by bacteria (**B**). (**A**) AgNPs can cross the plasma membrane by diffusion (**1**), endocytotic uptake (**2**,**3**), and disruption of membrane integrity (**4**). (**B**) AgNPs permeate the cell walls of gram-negative and gram-positive bacteria. Reproduced from [36], MDPI under the Creative Commons Attribution License.

Conventional antibiotics such as tetracycline and streptomycin have been widely used to inhibit bacterial infections. However, these antibacterial agents are ineffective to inhibit multidrug-resistant bacterial strains. This is because such bacteria are getting more resistant to biocidal action of antibiotic molecules. Therefore, it deems necessary to develop biomaterials that target drug-resistant bacteria. In this respect, AgNPs with large surface areas can provide a better contact for interacting with bacteria compared to conventional silver microparticles. Nanosilver in the form of colloidal silver and silver nitrate have been used for more than 100 years as a biocidal agent in the United States [32]. AgNPs have been reported to be effective in killing both gram-negative and gram-positive bacteria strains [23,33,34,35]. However, AgNPs are more effective in destroying gram-negative bacterial strains than gram-positive because gram-positive bacteria have one cytoplasmic membrane and a relatively thick cell wall comprising of several peptidoglycan layers (20–80 nm), while gram-negative bacteria have an external layer of lipopolysaccharide (LPS) followed by a thin layer of peptidoglycan and an innermost plasma membrane (Figure 2) [36,37]. AgNPs can also eliminate multidrug resistant (MDR) bacteria by interfering with their defense mechanisms. They can be used alone or in combination with antibiotics [15,38,39,40]. Gurunathan et al. indicated that biosynthesized AgNPs are very effective in killing MDR bacteria *Prevotella melaninogenica* and *Arcanobacterium pyogenes* [38]. Katva et al. reported that AgNPs combined with gentamicin and chloramphenicol exhibit a better antibacterial effect in *Enterococcus faecalis* than both antibiotics alone. *Enterococcus faecalis* is a MDR bacteria which is resistant to a wide range of antibiotics [40]. The antibacterial activity of AgNPs is known to be shape-, size-, charge-, and dose-dependent [15,41,42,43]. Xia et al. reported that a series of Ag nanocrystals with controlled shapes and sizes can be synthesized from silver salts by using different combinations of seeds and capping agents [44]. Recently, Hosseinidoust et al. reported a one-pot green synthesis of colloidally stable AgNPs having triangular, hexagonal and dendritic shapes without using toxic chemicals and seeds [45].

In general, AgNPs act like a double-edged sword with beneficial and harmful effects, i.e., they can eliminate bacteria but also induce cytotoxicity. Due to the versatility of AgNPs in many consumer and health products, there is growing public concern about the risk of using those products because AgNPs may pose potential health hazards. Furthermore, extensive application and production of AgNPs would increase their release into aquatic environments such as rivers and lakes. For instance, AgNPs can be released from antimicrobial fabrics into water during washing, thereby polluting groundwater environment [23,46,47]. Once AgNPs enter freshwater environment, they usually oxidize into Ag^+^ ions that are toxic to aquatic organisms. Moreover, ionic silver is immobilized to a large extent as a sparingly soluble salt like AgCl or Ag_2_S [23]. By accumulating in aquatic organisms, AgNPs can enter the human body through the food chain. However, little is known about the long-term safety and toxic effects of AgNPs in the aquatic environment. Humans can be exposed to AgNPs via several routes including inhalation, oral ingestion, intravenous injection, and dermal contact. AgNPs then enter human cells either by endosomal uptake or by diffusion (Figure 2) [36]. The American Conference of Governmental Industrial Hygienists (ACGIH) has established threshold limit values for metallic silver (0.1 mg/m^3^) and soluble compounds of silver (0.01 mg/m^3^). As recognized, extended exposure to Ag through oral and inhalation can lead to Argyria or Argyrosis, i.e., chronic disorders of skin microvessels and eyes in humans [23,48]. In vitro cell culture studies have indicated toxic effects of AgNPs in immortal human skin keratinocytes (HaCaT), human erythrocytes, human neuroblastoma cells, human embryonic kidney cells (HEK293T), human liver cells (HepG2), and human colon cells (Caco2) [49,50,51,52,53,54,55]. In vivo animal studies have revealed toxic effects of AgNPs in rodents by accumulating in their liver, spleen, and lung [56,57]. Similarly, AgNPs-mediated cytotoxicity in mammalian cells [55,58,59,60,61,62] depends greatly on the nanoparticle size, shape, surface charge, dosage, oxidation state, and agglomeration condition as well as the cell type. This article provides a state-of-the-art review on the recent development in the synthesis of AgNPs, their antibacterial activity, and cytotoxic effects in mammalian cells, especially in the past five years. Proper understanding of the interactions between AgNPs and mammalian cells is essential for the safe use of these nanoparticles. This knowledge enables scientists to develop functional AgNPs with improved biocompatibility to mammalian cells for combating MDR bacteria.

## 2. Synthesis of AgNPs and Their Polymer Nanocomposites

AgNPs can be prepared from physical, chemical, and biological routes [63]. The physical route has a distinct advantage in forming high-purity AgNPs. However, it is a low yield process, which often requires high temperature and power consumption, thereby limiting their application in the industrial sector. In contrast, AgNPs can be produced in large quantities through chemical and biological routes due to their cost-effectiveness. In this respect, we focus mainly on nanoparticle fabrication using chemical and biological routes in this section. The physical synthesis process generally employs thermal, laser, or arc discharge power to form AgNPs with nearly narrow size distribution. In this context, evaporation condensation and laser ablation strategies are commonly adopted to form AgNPs. The former approach is carried out by simply placing a source metal, i.e., silver target inside the tube furnace, and then vaporized silver into a carrier gas under atmospheric pressure at high temperatures. The vapor is then condensed to form nanoparticles. The drawbacks of this approach include high power consumption and long heating time to reach thermal stability. Jung et al. modified this process by using a small ceramic heater with a local heating area for vaporizing silver metal [64]. The temperature gradient in the vicinity of the heater surface is very steep, such that the vapor can cool at a rapid rate, thereby condensing into spherical AgNPs with diameters of 6.2–21.5 nm without agglomeration. Alternatively, laser ablation can be used to synthesize AgNPs from a silver target placed in a solution under laser beam irradiation. The nanoparticle size of colloids depends on the laser wavelength, ablation time, and duration of laser pulses [65,66,67]. The limitation of this process is the high cost of laser facility.

### 2.1. Wet Chemical Route

The wet chemical route is by far the most economical and commonly used process for preparing nanosilver colloidal dispersions in water or organic solvents. This route includes chemical reduction, microwave-assisted synthesis, microemulsion, photoreduction, electrochemical approach, etc. [41,42,43,62,68,69,70,71,72]. Among these, chemical reduction is a relatively simple, high yield and cost-effective process through the chemical reduction of silver salt in water or an organic solvent to form a colloidal suspension. This strategy requires silver salt, reducing agent and stabilizing/capping agent. Silver nitrate is the most widely used silver salt precursor. Sodium borohydride (NaBH_4_), ascorbic acid, glucose, hydrazine, sodium citrate, and ethylene glycol (EG) are typical reductants for reducing silver ions [71,72,73,74]. Sodium borohydride is a strong reductant for forming fined and monodispersed AgNPs. Weak reducing agents such as sodium citrate, ascorbic acid, and glucose lead to the formation of relatively large AgNPs, having a wider size distribution; sodium citrate plays the role of a reducing and a stabilizing agent [73]. As recognized, colloidal AgNPs tend to contact or link with each other to form aggregates as a result of the attractive Van der Waals forces. The agglomeration of colloidal AgNPs can be prevented by the use of stabilizing agents. The stabilizing effect of polymer-based capping agents or non-ionic surfactants of AgNPs suspensions is based on the charge repulsion or steric hindrance to counteract the van der Waals attraction between colloidal nanoparticles. As such, electrostatic or steric stabilization of colloidal AgNPs is achieved through adsorption of macromolecules or organic compounds to the surfaces of the nanoparticles. Citrate-capped AgNPs exhibit negative surface charge due to the carboxylic moiety of citrate. This leads to electrostatic repulsion between AgNPs, thereby preventing agglomeration of nanoparticles [71,75]. Typical polymer-based capping agents are polyvinyl alcohol (PVA), polyvinylpyrrolidone (PVP), polyethylene glycol (PEG) and polysaccharides, while non-ionic surfactants including Brij, Tween, and Triton X-100 are employed to stabilize AgNPs during the formation process [76,77]. These stabilizers also play important roles in controlling the size and shape of AgNPs. The properties and bactericidal activities of AgNPs are greatly influenced by their size and shape. Thus, researchers have spent much effort on the size- and shape-controlled synthesis of AgNPs by using different capping agents. Organic solvents have some advantages over aqueous solutions such as high yield and narrow particle size distribution. In certain cases, solvent itself can also serve as a reducing agent [78]. As an example, the polyol process utilizing EG as both solvent and reductant in the presence of PVP at 160 °C is known to be effective to reduce silver nitrate to yield AgNPs [79,80]. In addition, microwave irradiation is more energy efficient than thermal heating in the polyol process. Microwave irradiation offers fast and homogeneous heating of the reaction medium, typically in a time period of few seconds. As such, it provides uniform nucleation and growth conditions for AgNPs [81].

**Figure 3:**
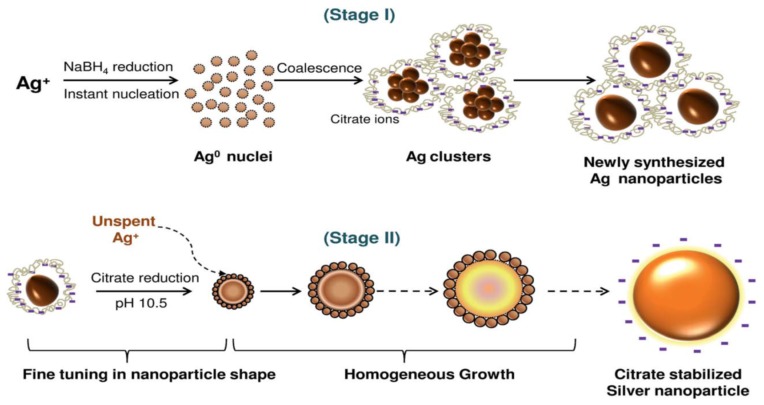
Schematic representation of size-controlled AgNPs synthesis employing the co-reduction strategy. Reproduced from [41], the Royal Society of Chemistry.

**Figure 4:**
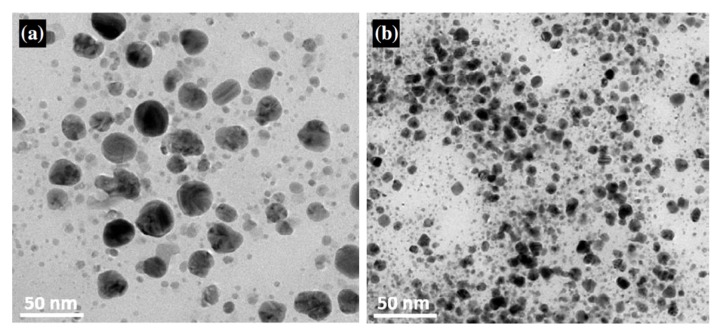

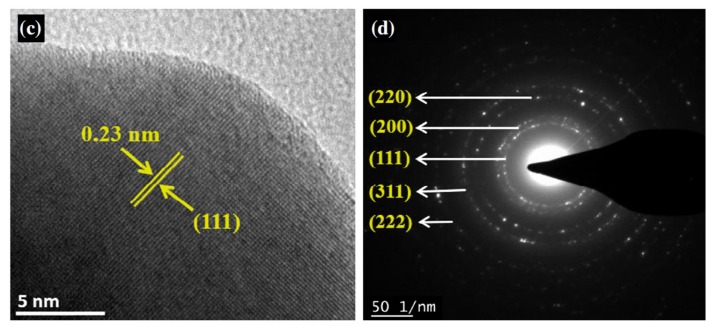
TEM images of AgNPs formed at (**a**) pH 6, and (**b**) pH 12. (**c**) High-resolution TEM image and (**d**) selected area electron diffraction pattern of AgNP. Reproduced from [82] with permission of Elsevier.

The formation of colloidal solutions via silver salt reduction generally proceeds through two stages: nucleation and subsequent growth. The nucleation stage involves the reduction of Ag^+^ to Ag^0^ atoms, and subsequent aggregation of Ag^0^ atoms to form clusters, (Ag^0^)_n_, according to the reactions: nAg^+^ → nAg^0^ → (Ag^0^)_n_, or via a stepwise reduction mechanism: Ag^+^ → Ag^0^ → Ag_2_^+^ → Ag_4_^+^ →⋯→ (Ag^0^)_n_. When the critical size is reached, nanoparticle nuclei will grow accordingly [74]. These stages can be manipulated by monitoring reaction conditions such as pH, temperature, precursors, reductants and stabilizers. Thus, the shape, size and size distribution of AgNPs can be controlled by varying the reaction parameters. As mentioned above, sodium citrate is a weak reductant leading to the formation of polydispersed AgNPs. Agnihotri et al. demonstrated that the size of AgNPs can be controlled by using a co-reduction approach, i.e., NaBH_4_ acts as a primary reductant, while citrate ions act as both a secondary reductant and a capping agent (Figure 3) [41]. Recently, Ajitha et al. fabricated PVA-capped AgNPs by reducing silver nitrate with NaBH_4_ in ethanol solvent in the presence of PVA [82]. The pH of the suspensions was further regulated by adding sodium hydroxide. They reported that the size of AgNPs decreases with increasing pH of the solutions, as revealed by transmission electron microscope (TEM) images (Figure 4a,b). The lattice spacing (i.e., 0.23) of AgNP (111) plane in high-resolution TEM image (Figure 4c), and the presence of rings in selected area electron diffraction pattern (Figure 4d) reveal the formation of nanocrystalline silver.

The size, geometry, morphology and homogeneity of AgNPs can also be manipulated by means of microemulsion technique. Several surfactants can be used to form microemulsion, including cationic cetyltrimethylammonium bromide (CTAB), anionic sodium dodecyl benzene sulfonate (SDBS) and sodium dodecyl sulfate (SDS), and nonionic Triton X-100 [83,84]. Water-in-oil (W/O) microemulsions or reverse micelles are often employed to prepare metallic nanoparticles [85,86,87]. They consist of nanosized water droplets suspended in a continuous oil phase and stabilized by surfactant molecules located at the oil/water interface. Thus, reactants can be introduced into water droplets acting as microreactors, leading to the formation of nanoparticles with uniform size distributions. For preparing metallic nanoparticles, two W/O microemulsions containing respective metal salt and reductant are mixed together to produce nanoparticles. As a result, an exchange of the reactants between micelles takes place during the collisions of water droplets, leading to coalescence, fusion, and mixing of the reactants. The size and shape of nanoparticles depend on the size and shape of water droplets, and the type of surfactant employed [88]. For instance, silver nitrate solubilized in the water core of one microemulsion can act as a source of silver ions, hydrazine hydrate dispersed in the water core of another microemulsion as a reducing agent, cyclohexane as the continuous phase, SDS as the surfactant, and isoamylalcohol as the cosurfactant [85]. It is noted that microemulsion process needs large quantities of surfactants and organic solvents, thus increasing the cost of production and polluting the environment. As the surfactants and solvents are mostly toxic [83], recent attention is paid to the use of natural plant extracts as the oil phase and reductant, or microorganisms as biosurfactants to produce AgNPs [86,87].

Similarly, organic solvents and reductants used in the chemical reduction process, such as *N*,*N*-dimethylformamide (DMF), dimethyl sulfoxide (DMSO), hydrazine, and sodium borohydride are toxic chemicals. This toxicity poses a threat to the environmental and living organisms. In this respect, photoreduction of silver salt through ultraviolet (UV) light without using toxic reductants and solvents can be used to form AgNPs. Thus, this is a simple and ecofriendly strategy to produce AgNPs [89,90,91]. For example, AgNPs can be synthesized from [Ag(NH_3_)_2_]^+^ aqueous solution under UV irradiation using PVP as both reducing and stabilizing agents The resultant particles exhibit nano-size (4–6 nm), monodisperse and uniform size distribution [90]. Lu et al. prepared AgNPs with and without PVP as a surface capping agent by employing photochemical synthesis [89].

### 2.2. Biological Route

In recent years, green synthesis has opened up a new direction for forming AgNPs with different sizes and shapes without using toxic reductants and stabilizers [92]. The advantages of green synthesis over chemical and physical routes including ecofriendly, cost-effective, natural abundance and easy to scale-up for mass production of nanoparticles. In this respect, bacteria, plant extracts, fungi, polysaccharides and their derivatives can be used as the reducing agents and stabilizers. Biosynthesis of AgNPs using certain bacterial strains shows little industrial and medical applications because it may pose health risk to humans. Therefore, plant extracts such as leaves, stems, fruits and seeds are attractive reagent materials to form green AgNPs. Natural plants generally contain carbohydrates, fats, proteins, nucleic acids and pigments that can act as effective reducing agents and stabilizers for silver ions. In particular, polysaccharides possess many functionalities such as hydroxyl groups and a hemiacetal reducing end that play crucial roles in both the reduction and the stabilization of metallic nanoparticles [93]. Polysaccharides and their derivatives include chitosan, cellulose, starch, hyaluronic acid and heparin. In addition, whole leaf extracts are rich in polyphenols such as flavonoids, which are effective reductants for fabricating AgNPs [93]. Furthermore, parameters like nature of plant extract, pH, and reaction time greatly affect the size, shape, and morphology of green AgNPs. Extensive studies have been conducted by researchers on the biosynthesis of AgNPs, especially using plant leaves [94,95,96,97,98,99,100,101,102].

**Figure 5:**
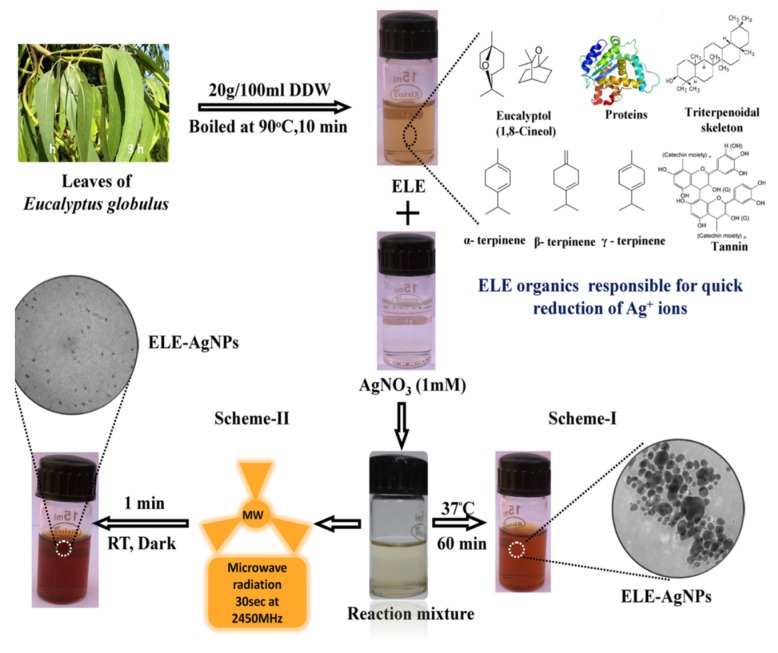
Graphical representation of AgNPs synthesis with Eucalyptus globulus leaf extract (ELE) and silver nitrate depicting scheme-I (without microwave treatment) and scheme-II with microwave irradiation. Reproduced from [106] with permission of Public Library of Science.

One main drawback of biosynthesis of AgNPs is the long reaction time. Microwave-assisted synthesis has attracted a great interest in biosynthesis because it can increase the reaction rate and product yield compared to conventional thermal heating [103]. Several successful reports relating microwave-assisted biosynthesis of AgNPs can be found elsewhere [104,105,106,107,108,109,110]. Peng et al. synthesized spherical AgNPs with sizes of 8.3–14.8 nm in an aqueous medium using bamboo hemicelluloses as a stabilizer and glucose as a reductant under microwave irradiation [104]. Ali et al. biosynthesized AgNPs with Eucalyptus globulus leaf extract (ELE) and AgNO_3_ with and without microwave irradiation (Figure 5) [106]. The solution mixture was heated with microwave radiation at 2450 MHz for 30 s. The size of biosynthesized AgNPs ranged from 1.9–4.3 and 5–25 nm, with and without microwave treatment, respectively. The size of microwave-treated AgNPs (scheme-II) was smaller than that formed by conventional process at room temperature (scheme-I), because the extent of nucleation and capping was faster with microwave heating than synthesis at 37 °C. By heating with microwave radiation for 60 s, the reaction rate increased such that more nucleation sites were formed due to the availability of −OH ions at high temperatures. Organic constituents such as flavanoids and terpenes in ELE were reported to be surface active molecules to stabilize AgNPs [106].

### 2.3. AgNP-Polymer Nanocomposites

As aforementioned, AgNPs find extensive applications in wound dressings, food packaging films or containers, antimicrobial fabrics, clinical scaffolds, etc. For those applications, AgNPs are typically embedded in the polymer matrix to form polymer nanocomposites. In this respect, polymers with high flexibility are ideal materials for protecting nanoparticles from mechanical damage [111,112]. A typical example is Acticoat™ Flex 3 dressing for burn care, i.e., a flexible polyethylene cloth coated with AgNPs at a concentration between 0.69 and 1.64 mg/cm^2^. This dressing can release a sustained amount of nanocrystalline silver and silver ions to the wound area [27]. Moreover, immobilization of AgNPs onto textile fibers can also impart colors to the fabrics in addition to antimicrobial features due to the surface plasmon resonance effect of silver. This avoids the use of toxic agents to fix colorants to the textiles [30,31,113]. In this respect, AgNPs function as a simultaneous colorant and antimicrobial agent for fabrics [113]. Traditional polymer microcomposites are usually employed as components for structural engineering applications due to their light weight and low cost [114,115,116,117,118]. However, polymer microcomposites are reinforced with fillers of micrometer scale at loadings ≥ 30 wt% to achieve desired mechanical performance. The additions of microfillers with high loading levels lead to poor processability and low ductility of resultant composites. On the contrary, polymer nanocomposites only require low nano-filler loadings (say 0.1–1 wt%) for electronic, medical, and structural engineering applications. These nanocomposites have been prepared by means of solution mixing, electrospinning, extrusion or injection molding [119,120,121,122].

The fabrication techniques for AgNP-polymer nanocomposites vary from one to another depending on their specific intended applications. Water-soluble polymers such as PVA, PEG, and polyacrylic acid (PAA) are commonly used as hydrogels in tissue engineering. Hydrogels are crosslinked polymer networks that swell in water. Polymeric hydrogels can be simply prepared by freeze-thawing cyclic processing without the utilization of chemical crosslinkers [123,124]. The freezing step at low temperatures induces a liquid–liquid phase separation due to the formation of ice crystals that expel polymer chains. This creates polymer-poor and polymer-rich phases accordingly. Upon thawing, the ice crystals melt, leaving behind pores between cross-linked polymer regions. Repeated freeze-thawing cycles of the polymer solution lead to the formation of crystallites that act as cross-linking sites, so a hydrogel with a high swelling capacity can be produced [125]. AgNP-polymer composite hydrogels can be prepared by introducing pre-formed AgNPs into a water-soluble polymer matrix, or the formation of AgNPs in-situ through chemical reduction in aqueous polymer solution. In the latter case, metal salt is dissolved in aqueous polymer solution, followed by in situ reduction with sodium borohydride and freeze-thawing cycles [126,127].

In the case of antimicrobial fabrics, the dip-coating method is commonly adopted in which the fabrics are immersed in a silver salt solution followed by chemical reduction [128,129,130,131,132,133]. In some cases, UV- or microwave-radiation is used to speed up the reaction rate and to control the size of AgNPs on fabric fibers [134,135]. Babaahmadi and Montazer reported one-step in situ synthesis of Ag NPs on polyamide (nylon) fabrics through the reduction of silver nitrate with stannous chloride (SnCl_2_) using CTAB as a stabilizer [129]. Montazer et al. then prepared AgNPs using [Ag(NH_3_)_2_]^+^ complex with PVP as a reducing/stabilizing agent under UV irradiation. These nanoparticles were finally deposited onto nylon fabric using a simple dip-pad technique [134]. In a later study, they introduced AgNPs within the polymeric chains of polyamide-6 fabric by using [Ag(NH_3_)_2_]^+^ complex [133]. The silver complex was reduced directly by the functional groups of polyamide chains without using any reductants. Moreover, nitrogen atoms of polyamide chains can stabilize AgNPs through coordination between the amide groups and silver.

As recognized, AgNPs can be deposited on fabrics more effectively by using plasma treatment through the creation of active groups on fabric fibers [136]. Recently, Zille et al. carried out dielectric barrier discharge (DBD) plasma treatment on polyamide 6,6 (PA 6,6) fabrics, followed by coating with colloidal AgNPs of different sizes [137]. Plasma pre-treatment promoted formation of oxygen species on fabric fibers, facilitating both ionic and covalent interactions between the oxygen species and AgNPs on the fibers. This led to the deposition of fined AgNPs on PA 6,6 fibers. Ilic et al. employed radio frequency (RF) plasma to etch the fibers of polyester fabrics in order to enhance binding efficiency of colloidal AgNPs onto polyester fibers [138]. They found that plasma treatment positively affected the loading of AgNPs on the fibers and antibacterial activity of polyester nanocomposite materials.

## 3. Antibacterial Activity

AgNPs are well known for their remarkable antimicrobial properties against various pathogens including bacteria, fungi, and viruses. However, the mechanisms responsible for the bactericidal effect of AgNPs remain unclear. There is an ongoing debate over whether AgNPs or silver ions exert a cytotoxic effect on microorganisms. The killing effect of AgNPs have been proposed to be associated with a direct contact of nanoparticles to the bacterial cell wall, followed by penetrating into cytoplasm. Direct contact of AgNPs with large surface areas on a bacterial cell wall could lead to membrane damage, resulting in the leakage of cellular contents and eventual cell death [139,140,141,142]. In particular, AgNPs with sizes below 10 nm are more toxic towards bacteria [141]. By penetrating into the cytoplam, AgNPs may interact with biomolecules such as proteins, lipids, and DNA. In certain cases, AgNPs can interact with the respiratory enzyme system, thereby generating reactive oxygen species (ROS) such as hydrogen peroxide (H_2_O_2_), hydroxyl (OH^–^) and superoxide (O_2_^−^) radicals that induce oxidative stress and damage to proteins and nucleic acids [38]. Herein, we present literature reports that support this mechanism. Earlier work by Sondi and Salopek-Sondi indicated that the accumulation of AgNPs (12 nm) on the cell wall of *Escherichia coli* (*E. coli*) leads to the formation of pits [139]. Those pits cause a loss of outer membrane integrity, resulting in the release of LPS molecules and membrane proteins, and causing eventual cell death. Morones et al. reported that AgNPs (1–10 nm) anchor to the cell wall of *E. coli* and disturb its normal function such as permeability and respiration. The nanoparticles also penetrate into cytoplasm and interact with protein and DNA leading to cellular death [143]. Moreover, AgNPs can also release silver ions, resulting in further cell damage. Recently, Gahlawat et al. also demonstrated that AgNPs (10 nm) attach to the cell wall of cholera, thereby interrupting permeability and metabolic pathways of the cell and causing cell death [140].

**Figure 6:**
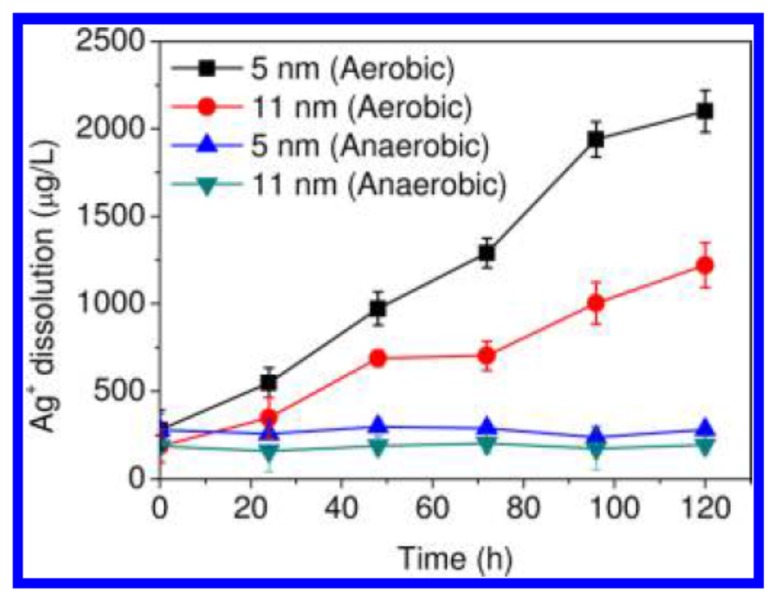
Dissolved Ag^+^ concentration vs. air exposure time for PEG-AgNPs with sizes of 5 and 11 nm under aerobic conditions. No Ag^+^ ions can be detected (<1 μg/L) under anaerobic conditions. Reproduced from [147] with permission of the American Chemical Society.

**Figure 7:**
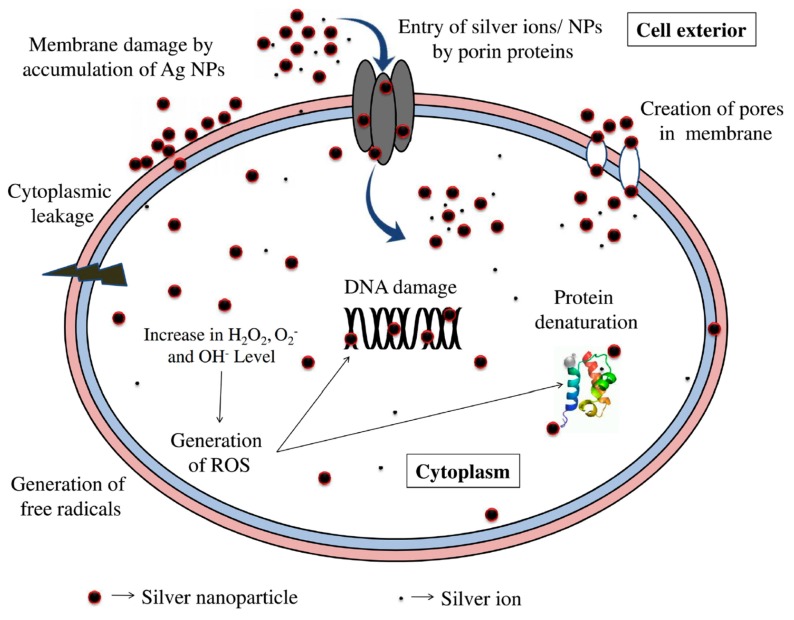
Bactericidal mechanisms of AgNPs due to their direct contact with the bacterial cell wall and the release of silver ions. Reproduced from [154] with permission of Elsevier.

On the contrary, the cytotoxic effect of AgNPs against bacteria may result from the oxidative dissolution of Ag^+^ ions from AgNPs. As is known, metals can be chemically oxidized in aqueous solutions to give metallic ions [144,145,146]. In this respect, AgNPs can be oxidized in aerated aqueous solutions to yield Ag^+^ ions [23]. Xiu et al. demonstrated that silver ions released from AgNPs in aerobic conditions are fully responsible for antibacterial activity (Figure 6) [147]. Small AgNPs (ca 5 nm) can release more Ag^+^ ions than large AgNPs (11 nm) under aerobic conditions due to their higher surface-to-volume ratio. In an anaerobic environment, very little Ag^+^ ions are released, so AgNPs themselves are non-toxic to bacteria. The antimicrobial action of Ag^+^ ions is closely related to their interaction with thiol (sulfhydryl) groups [148]. Thus, Ag^+^ ions can react with the -SH groups of cell wall-bound enzymes and proteins, interfering with the respiratory chain of bacteria and disrupting bacterial cell wall. Moreover, those ions released from AgNPs can penetrate the cell wall into the cytoplasm, thereby degrading chromosomal DNA [149], or reacting with thiol groups of the proteins in cytoplasm. Consequently, DNA loses its replication ability and the proteins essential to the ATP production becomes inactivated. In general, smaller silver particles can enter the cytoplasm more easily than larger particles [150]. AgNPs that have penetrated inside the cell can also release Ag^+^ ions, thereby generating free radicals and oxidative stress accordingly. Bondarenko et al. reported that there exists a synergistic effect between these two mechanisms for antibacterial activity. Direct cell−nanoparticle contact promotes the release of silver ions from AgNPs, thereby enhancing the amount of cellular uptake of particle-associated Ag^+^ ions [151]. Ivask et al. demonstrated that positively charged ions released from AgNPs tend to interfere with the normal function of the bacterial electron transport chain of *E. coli*, thereby facilitating the formation of reactive oxygen species (ROS) [152]. ROS generation is mostly responsible for the bacterial death because it facilitates lipid peroxidation, but inhibits ATP production and DNA replication. Elevated ROS levels in bacterial cells can result in oxidative stress [153]. Figure 7 shows a schematic representation of bactericidal effects due to AgNPs- induced membrane damage and silver ion release from the nanoparticles, or the combination of these two effects [154]. These mechanistic effects can be summarized into an initial attachment of AgNPs or Ag^+^ ions to the bacterial cell wall, their subsequent penetration inside the cell, followed by ROS and free radical generation, DNA damage and protein denaturation.

**Figure 8:**
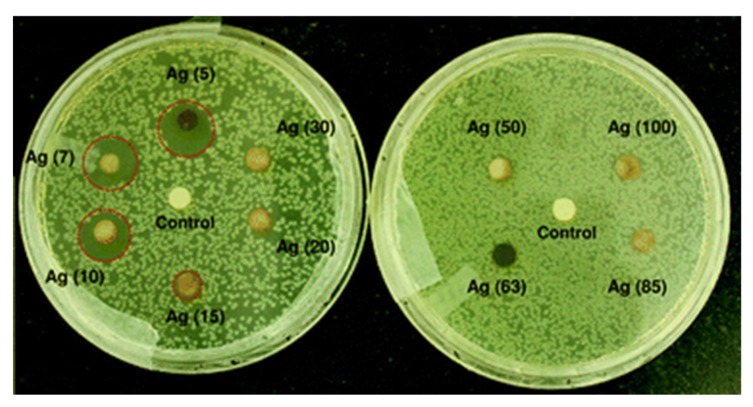
Disk diffusion assay results for AgNPs of various sizes against *E. coli*. The zone of inhibition is highlighted with a dashed circle indicating a noticeable antibacterial effect. Reproduced from [41], the Royal Society of Chemistry.

The antibacterial efficacy of AgNPs relates to the kinds of pathogenic bacteria. Gram-negative bacteria are generally more prone to Ag^+^ invasion than gram-positive bacteria due to the difference in their cell wall structures (Figure 2). As aforementioned, gram-positive bacteria possess a very thick cell wall containing many peptidoglycan layers, thereby serving as a barrier for Ag^+^ ions penetration into the cytoplasm. However, gram-negative bacteria only have a single peptidoglycan layer, thus Ag^+^ ions can easily damage the cell wall. The bactericidal effects of AgNPs also depend on the nanoparticle characteristics, including the size, shape, surface charge, dose and particle dispersion state [41,42,43,133,134,135,136,137,138,139,140,141,142,143,144,145,146,147,148,149,150,151,152,153,154,155,156,157,158,159,160,161]. Generally, well-dispersed AgNPs in physiological solutions exhibit greater bactericidal efficacy than agglomerated AgNPs. Moreover, the killing effect of AgNPs against gram-negative and gram-positive bacteria increases with decreasing particle size. Lu et al. prepared AgNPs of different sizes (~5, 15 and 55 nm) using a simple reduction method and found that AgNPs (5 nm) exhibited the highest antibacterial activity against oral bacteria [157]. Agnihotri et al. synthesized AgNPs of various sizes, i.e., 5, 7, 10, 15, 20, 30, 50, 63, 85 and 100 nm, and reported that AgNPs with sizes below 10 nm exhibited the best antibacterial activity for *E. coli* than larger nanoparticles (Figure 8) [41].

**Figure 9:**
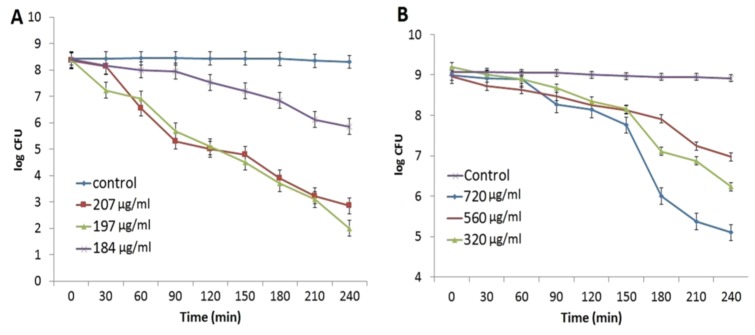
Killing kinetics of *K. pneumoniae* AWD5 exposed to (**A**) spherical AgNPs at concentrations of 184–207 μg/mL and (**B**) rod-shaped AgNPs at 320–720 μg/mL. Results were expressed as means ± SD; *n* = 3. *p* < 0.05 was considered statistically significant. Reproduced from [161], Nature Company under the Creative Commons Attribution License.

AgNPs can be produced in different shapes depending upon synthesized conditions [44,45,72]. Accordingly, AgNPs exhibit shape-dependent efficacy of bactericidal activities. Pal et al. reported that AgNPs with the same surface areas but different shapes exhibit dissimilar antibacterial activity. They found that truncated triangular silver nanoplates with a {111} lattice plane as the basal plane exhibit the strongest biocidal effect, compared with spherical and rod-shaped nanoparticles [160]. This is because the reactivity of silver is favored by a high-atom-density {111} plane. From the disk-diffusion tests, the bactericidal efficacy against *E. coli* of 10^7^ CFU/mL takes the following order: truncated triangular > spherical > rod-shaped AgNPs. Very recently, Archaya et al. investigated the bactericidal effects of spherical and rod-shaped AgNPs against gram positive (*S. aureus*, *B. subtilis*) and gram negative (*E. coli*, *K. pneumoniae AWD5*, *P. aeruginosa*) bacterial strains [161]. Among these strains, *Klebsiella pneumoniae* can cause pneumonia, bloodstream infection and wound infection. *Klebsiella* bacteria show resistance to antibiotics and pose serious threat to human health, as outlined by the World Health Organization [162]. Their results indicated that the bactericidal activity of both spherical and rod-shaped AgNPs is dose- and time- dependent. Spherical AgNPs are more effective than rod-shaped AgNPs in killing *Klebsiella* bacteria (Figure 9).

From the literature, the positive charge of the Ag^+^ ions is critical for their bactericidal activity through electrostatic attractions between the negatively charged cell wall of the bacteria and positively charged Ag^+^ ions [37]. The carboxyl, phosphate, hydroxyl, and amine groups associated with the thick peptidoglycan layer of the cellular wall of gram-positive bacteria render them with a negative charge. Similarly, those functional groups associated with LPS in the outer membrane confer an overall negative charge to the gram-negative cell wall [163]. As stated, the bactericidal effect of AgNPs is influenced by their surface charges. Thus, the capping agent and stabilizers used to prevent the aggregation of colloidal nanoparticles inevitably exert an influence on their surface charges [156,158,164]. Badawy et al. investigated the effect of surface charge of AgNPs capped with PVP, citrate (CT) and branched polyethyleneimine (BPEI) on bactericidal activity against the bacillus species. The BPEI-AgNPs are electrosterically stabilized through adsorption of the BPEI polyelectrolyte containing amine groups, which ionize in the solution to create charged polymers [164]. Electrosteric stabilization derives from both electrostatic repulsion and steric stabilization. Zeta potential of CT-AgNPs has a very negative value of −38 mV due to the carboxylic moiety of citrate. Consequently, there exists an electrostatic repulsion between the negatively charged CT-AgNPs and bacterial cell wall, thereby forming an electrostatic barrier that restrains the cell-particle interactions and thus reducing toxicity. The zeta potential of PVP-AgNPs is less negative, i.e., −10 mV, thus promoting cell-particle interactions and resulting in a higher toxicity than CT-AgNPs. The electrostatic repulsion changes to attraction by exposing bacteria to positively charged BPEI-AgNPs (+40 mV). In this case, BPEI-AgNPs interact strongly with the negatively charged moieties in the bacteria membrane (e.g., proteins) and induce changes in structural integrity of the bacteria cell wall, leading to the leakage of cytoplasmic contents and eventual cell death. These results reveal surface charge-dependent toxicity of AgNPs capped with different stabilizing agents on the *bacillus* species [164]. Thus, electrosterically coated BPEI-AgNPs exhibit a higher toxicity than electrostatically capped CT-AgNPs and sterically stabilized PVP-AgNPs. Similarly, Lee et al. demonstrated that PEI-AgNPs exhibit a positive zeta potential of +49 mV. PEI is a cationic polymer in which the amino groups provide AgNPs with a positive charge and stability against agglomeration [158]. Moreover, PEI-AgNPs show excellent bactericidal activity against *S. aureus and K. pneumoniae*. Recently, Abbaszadegan et al. studied the effect of surface charge of AgNPs on antimicrobial activity against gram-positive (*S. aureus*, *Streptococcus mutants*, *and Streptococcus pyogenes*) and gram-negative bacteria (*E. coli* and *Proteus vulgaris*) [156]. They indicated that positively-charged AgNPs exhibit the highest bactericidal activity against all bacterial strains. The negatively charged AgNPs have the least, while neutral AgNPs show intermediate antibacterial activity.

### 3.1. Biosynthesized AgNPs

**Figure 10:**
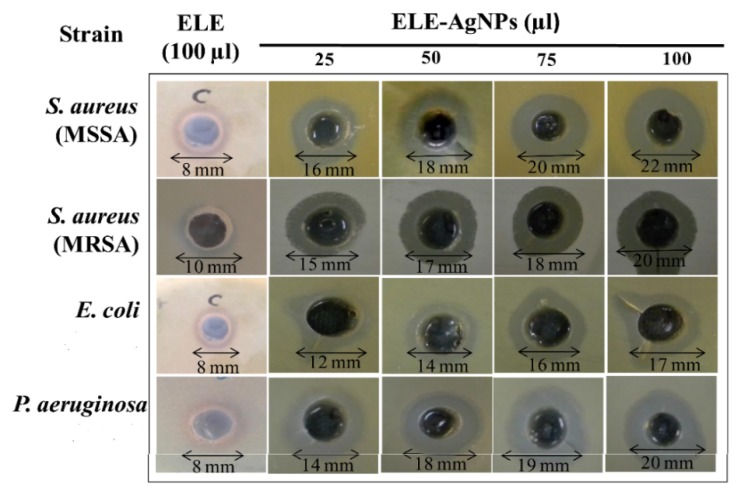
Assessment of antibacterial activity of ELE and ELE-AgNPs by disk diffusion assay. Reproduced from [106] with permission of Public Library of Science.

**Figure 11:**
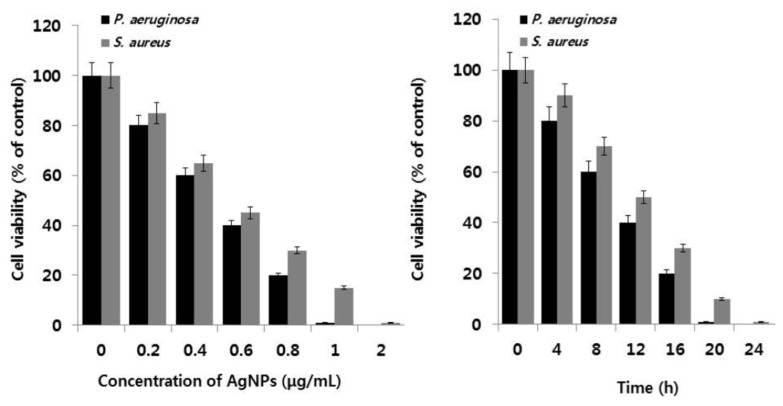
(**Left**): Effect of AgNPs concentration on bacterial cell viability. Bacterial survival was determined at 24 h based on a colony-forming unit (CFU) count assay. (**Right**): Time-dependent bactericidal effect of AgNPs on *P. aeruginosa* and *S. aureus*. Results were expressed as means ± SD; *n* = 3. *p* < 0.05 was considered statistically significant. Reproduced from [107], MDPI under the Creative Commons Attribution License.

**Figure 12:**
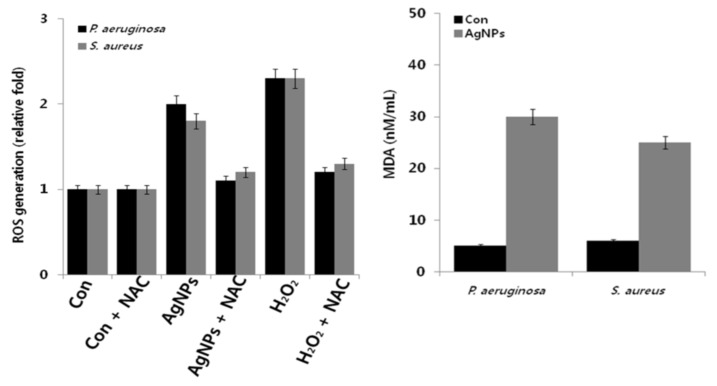
Effects of AgNPs on ROS (left panel) and MDA (right panel) levels. Results were expressed as means ± SD of *n* = 3; *p* < 0.05 was considered statistically significant as compared to control (con) groups. Reproduced from [107], MDPI under the Creative Commons Attribution License.

Ali et al. employed Eucalyptus globulus leaf extract (ELE) to stabilize colloidal AgNPs during synthesis. They reported that ELE-AgNPs are effective antibacterial and antibiofilm agents for gram-negative *P. aeruginosa* and *E. coli*, gram positive methylene resistant *S. aureus* (MRSA) and methylene sensitive *S. aureus* (MSSA) [106]. Figure 10 shows the disk diffusion assay results for these bacterial strains exposed to ELE-AgNPs with concentrations ranging from 25–100 μL. Gurunathan and coworkers carried out a comprehensive study on antibacterial activity of biosynthesized AgNPs prepared from quercetin against *S. aureus* and *P. aeruginosa* [107]. Several bioassays for detecting colony-forming unit (CFU), lactose dehydrogenase (LDH), ROS generation, malondialdehyde (MDA), glutathione (GSH), etc. were employed to assess antibacterial activity. The AgNPs exhibited a spherical feature with an average size of 11 nm. The minimum inhibitory concentrations (MICs) of AgNPs against *P. aeruginosa* and *S. aureus* were 1 and 2 µg/mL, respectively. The bactericidal effect of AgNPs on bacteria came from the generation of ROS and MDA, and the leakage of proteins and sugars in bacterial cells. Figure 11 shows the dose- and time-dependent bactericidal activities of AgNPs for both bacterial strains. Complete growth inhibition concentration and time were determined to be 1 μg/mL AgNPs and 20 h for *P. aeruginosa*, while those for *S. aureus* were 2 μg/mL and 24 h, respectively. Figure 12 shows the ROS and MDA levels of both bacterial strains treated with AgNPs. Apparently, high ROS and MDA levels were observed in both strains, leading to abnormal cell metabolism and function, and eventual cell death. MDA was a product of lipid peroxidation, thus serving as an indicator of oxidation stress. On the basis of these results, Gurunathan and coworkers demonstrated that biosynthesized AgNPs is an effective therapeutic agent for treating mastitis-infected goats in husbandry.

**Figure 13:**
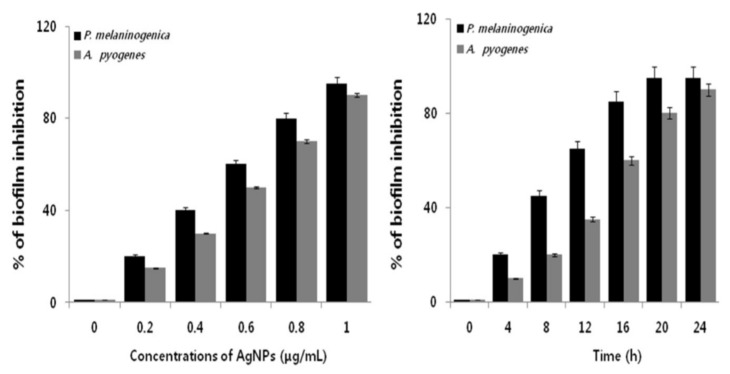
Anti-biofilm behavior of AgNPs on *P. melaninogenica* and *A. pyogenes*. (**Left**): Bacterial strains were treated with AgNPs of different concentrations. (**Right**): Bacterial strains were incubated with 0.8 and 1.0 μg/mL of AgNPs, respectively, for 24 h. *p* < 0.05 was considered statistically significant as compared to control groups. Reproduced from [38], MDPI under the Creative Commons Attribution License.

With the increasing use of antibiotics in animal husbandry, bacteria have developed resistance to antibiotics that pose serious threats to human health. The creation of multi-drug resistant bacteria is increasing at an alarming rate. In this respect, AgNPs appear to be a promising therapeutic agent against microbial pathogens in husbandry, especially for bacteria with antibiotic resistance. Very recently, Gurunathan et al. synthesized AgNPs (10 nm) using apigenin as a reducing and stabilizing agent [38]. They reported that as-synthesized AgNPs were very effective in eliminating multidrug resistant bacteria *Prevotella melaninogenica* and *Arcanobacterium pyogenes*. From the cell viability assay, antibacterial activity of AgNPs was dose-dependent, and the minimum inhibitory concentration (MIC) values of AgNPs against *P. melaninogenica* and *A. pyogenes* were determined to be 0.8 and 1.0 μg/mL, respectively. The minimum bactericidal concentration (MBC) values of AgNPs against *P. melaninogenica* and *A. pyogenes* were 1.0 and 1.5 μg/mL, respectively. The antibacterial activity of AgNPs was derived from the ROS generation, LDH leakage and DNA damage in bacterial cells. Figure 13 depicts a typical anti-biofilm activity of AgNPs on *P. melaninogenica* and *A. pyogenes*.

### 3.2. Polymer-AgNPs Nanocomposites

#### 3.2.1. Nanocomposite Fabrics

As recognized, direct contact of AgNPs with human body inevitably leads to cytotoxicity and genotoxicity. Accordingly, it is necessary to immobilize AgNPs into polymeric materials to isolate them from the human body, and to control the release of Ag^+^ ions. In recent years, considerable attention has been paid to produce antimicrobial composite fabrics due to their attractive applications in healthcare and medical sectors [165]. However, the poor laundering durability of nanocomposite fabrics limits their applications as a result of weak bonding between the polymer fabrics and nanoparticles [166,167]. In particular, hospital textiles are laundered at elevated temperatures for many cycles to minimize the risk of contaminated linens and to prevent the spread of various diseases. Mechanical vibration coupled by high temperature conditions in washing machines can detach AgNPs from the fabrics. Some efforts have been taken by researchers to improve the adherence of AgNPs to fabric fibers including plasma deposition, choice of fabric materials, graft polymerization, etc. [134,168,169]. For instance, El-Rafie et al. applied 50 and 100 ppm biosynthesized AgNPs to cotton fabrics, and reported that the reduction rate of bacterial colonies was higher than 90% against *S. aureus* and *E. coli* before washing [167]. The antibacterial activity of the composite fabrics againsts both bacterial strains reduced by more that 40% after 20 washing cycles. The absence of chemical interactions between the AgNPs and cotton fibers led to poor binding of AgNPs to cotton fabrics. Consequently, some AgNPs were removed from the fabrics during washing cycles. Montazer et al. employed UV radiation to synthesize AgNPs using [Ag(NH_3_)_2_]^+^ and PVP. The as-sythesized PVP-AgNPs were deposited onto nylon fabrics using a dip-pad technique [134]. In the process, PVP-AgNPs with respective concentrations of 100 and 200 ppm were deposited onto nylon fabrics, and then exposed to *E. coli* and *S. aureus*. The bacterial reduction levels of unwashed and washed nanocomposite fabrics (10, 20, and 30 washes) were evaluated; the results are listed in Table 1. Apparently, nanocomposite fabrics exhibited good antibacterial property by eliminating *E. coli* up to 99% after 30 washes. This is because AgNPs coated on the fabric fibers were resistant to *E. coli* after repeated laundries. Moreover, capped PVP of the AgNPs can establish chemical linkages with polyamide chains of nylon, leading to a strong adherence of AgNPs to nylon. However, bactericidal activity of the fabric with 100 ppm AgNPs decreased slightly after repeated washing. The bacterial reduction percentage of *S. aureus* decreased slightly from 99.99% to 86.92% after 30 washes. This rate was acceptable for antimicrobial fabrics after several washing cycles.

**Figure 14:**
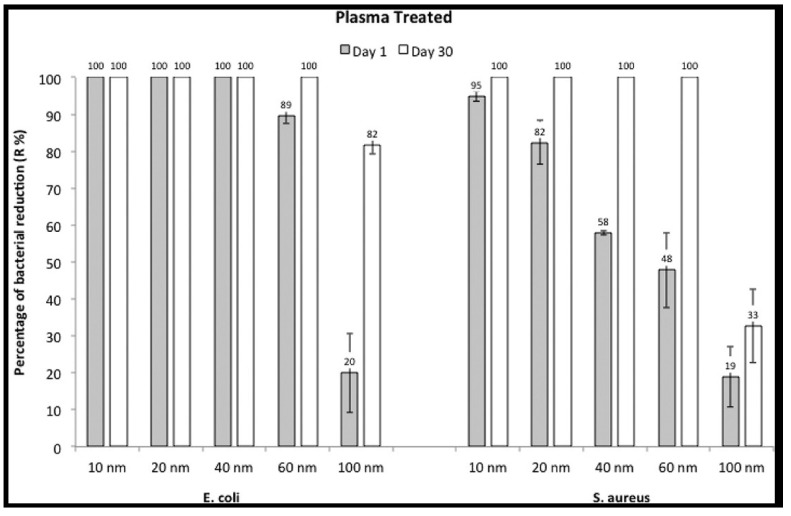
Percentage of bacterial reduction (*E. coli* and *S. aureus*) as a function of the size of AgNPs after exposure of 1 day and 30 days. Data are presented as mean values ± SD (*n* = 3). Reproduced from [137] with permission of the American Chemical Society.

As mentioned above, Zille et al. pretreated PA6,6 fabrics with DBD-plasma, followed by immersion in commercial colloidal AgNPs dispersions (10, 20, 40, 60 and 100 nm particle size) containing sodium citrate as a stabilizer to form PA6,6/AgNP composite fabrics [137]. Figure 14 shows the percentage of bacterial reduction vs. the size of AgNPs deposited on plasma-treated PA6,6 fabrics upon exposure to *E. coli* and *S. aureus* for 1 day and 30 days It can be seen that bacterial growth inhibition for S. aureus is size-dependent at day 1. The inhibition effect against *S. aureus* increases with decreasing nanoparticle size. The value decreases from 95% for the 10 nm-AgNPs, down to 19% for the 100 nm-AgNPs. The inhibition effect is associated with the release of Ag^+^ ions from the AgNPs in the solution during antimicrobial tests. Thus, more Ag^+^ ions are released from smaller AgNPs than larger nanoparticles due to their larger surface area-to-volume ratio. Upon exposure to *E. coli* at day 1, AgNPs with sizes of 10, 20 and 40 nm exhibit full bacteria inhibition, while AgNPs with sizes ≥60 nm show partial killing. Thus, AgNPs are more effective in eliminating *E. coli* due to their thin wall structure, as mentioned previously. At day 30, considerable amounts of Ag^+^ ions are released from the nanocomposite fabrics, and the growth of both *S. aureus* and *E. coli* are completely inhibited with the exception of 100 nm-AgNPs [137].

#### 3.2.2. Food Packaging Nanocomposite Films

**Figure 15:**
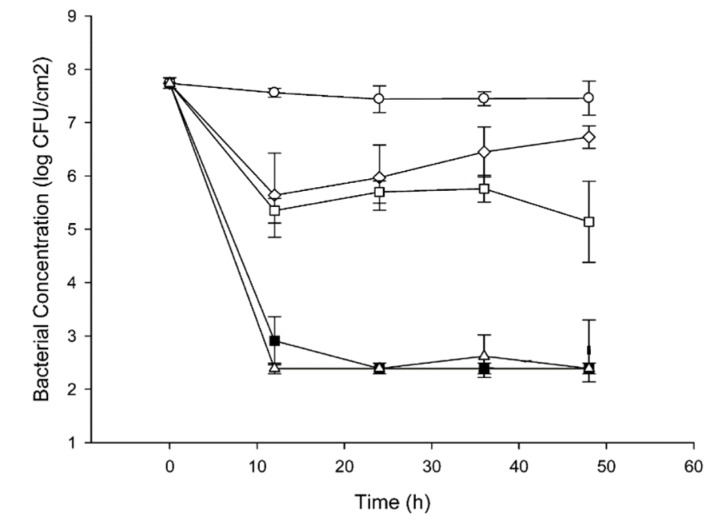
Viable counts in the challenge test on apple peels with *L. monocytogenes* versus silver nitrate aqueous solution (black square), EVOH (circle), and EVOH composite films with 0.1 wt% Ag^+^ (diamond), 1 wt% Ag^+^ (square), and 10 wt% Ag^+^(triangle). Reproduced from [175] with permission of the American Chemical Society.

In recent years, there has been a growing demand in food industries to develop antimicrobial food packaging films, bottles and containers to avoid microbial food spoilage and to extend or preserve shelf life. Food packaging is employed to protect foods, vegetables and fruits from environmental and bacterial contaminations to ensure their quality and food safety. Oxidation, microbial invasion, and metabolism are the main factors causing deterioration of foods and fruits during production, transportation, and storage [170]. Nowadays, AgNPs, silver nitrate and nanoclay are commonly used in the food packaging industry to resist microbial contamination and to improve barrier properties, thus prolonging shelf life and freshness of packaged foods and drinks [171,172,173,174]. As mentioned previously, colloidal nanosilver and silver nitrate have been used for more than 100 years in the United States [32]. Martınez-Abad et al. incorporated silver nitrate (0.1–10%) into ethylene-vinyl alcohol copolymer (EVOH) films and studied their antimicrobial behavior against *listeria monocytogenes* and *salmonella spp*. [175]. They employed the bacterial challenge test [176] to assess antimicrobial resistance of EVOH composite films against low protein food samples (lettuces, apple peels, and eggshells) and high protein food samples (chicken, marinated pork loin, and cheese) contaminated with those bacterial strains. Figure 15 shows representative viable bacterial counts on apple peels with *listeria monocytogenes*, and then treated with EVOH composite films containing 0.1, 1 and 10 wt% AgNO_3_. Composite film with 10% AgNO_3_ and control (silver nitrate solution) show a 4–5 log reduction in microbial population, while films with 0.1 and 1 wt% AgNO_3_ display little antimicrobial effect, i.e., a decrease of about 2 log bacterial counts after 24 h exposure. These results indicate that antimicrobial resistance of the composite films with 0.1 and 1 wt% AgNO_3_ on food samples are somewhat poorer than aqueous silver nitrate solution. This is due to the confinement of AgNO_3_ in the polymer matrix, thereby restricting the release of sufficient amounts of Ag^+^ ions to combat microorganisms. Thus, only composite film with high filler loading level, i.e., 10 wt% AgNO_3_ can achieve a similar antibacterial effect as silver nitrate solution.

In general, AgNPs exhibit a beneficial effect over silver nitrate salt in food packaging films because AgNPs allow a sustained release of Ag^+^ ions due to the size-related Ag^+^/Ag^0^ ratio on their surfaces [177]. In this respect, low AgNPs loadings are added in polymeric films to release sufficient Ag^+^ ions to ensure effective bactericidal activity [178]. More recently, Tavakoli et al. fabricated polyethylene (PE) films with 1, 2 and 3 wt% AgNPs using the extrusion process [173]. They reported that PE/AgNP packaging films decrease mold and coliform attack on walnuts, hazelnuts, almonds and pistachios for extended periods, thereby increasing shelf life and preserving the quality of nuts. The widespread use of polymer/AgNPs packaging films and containers in the food industry has resulted in increased concerns over the migration of AgNPs from the films or containers into foods. In this context, Huang et al. exposed commercial PE/nanosilver film bags to four kinds of food-simulating solutions, representing water, acid, alcohol and fatty foods, at 25–50 °C for 3 to 15 days, respectively. They found the migration of Ag^0^ from commercial PE/nanosilver films into food-stimulants on the basis of atomic absorption spectroscopic measurements [179]. It is considered that Ag^+^ ions are also released from nanocomposite films upon exposure to food-simulating solutions. Moreover, Ag^+^ ions are easily reduced to Ag^0^ in the presence of acid environments. Indeed, Echegoyen and Nerin reported the presence of both elemental Ag^0^ and Ag^+^ ions in commercial polyolefin packaging films and containers with nanosilver. Furthermore, microwave oven heating accelerates the migration of these species into stimulant solutions due to the structural modification of the polymer matrix [180].

#### 3.2.3. Nanocomposite Wound Dressings

**Figure 16:**
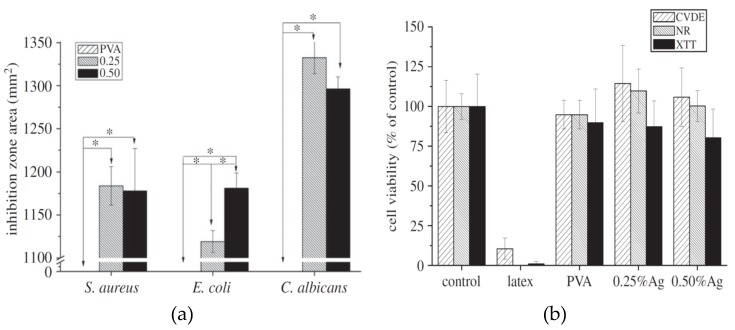
(**a**) Inhibition zones of all samples exposed to S. aureus, *E. coli* and *C. albicans*. There is a significant difference between the levels indicated by arrows, * *p* < 0.05. (**b**) Cell viability of mouse fibroblasts after 24 h incubation with nanocomposite hydrogels. CVDE (cell density), NR (membrane integrity assay) and XTT (mitochondrial activity). ‘Control’ is the negative control, whereas ‘latex’ is the positive control. Reproduced from [184] with permission of the Royal Society Publishing.

Hydrogels have been developed and used in the medical sector to enhance wound healing. They find attractive clinical applications due to their biocompatibility, high water content, and good absorption of wound exudates. By incorporating AgNPs or silver nitrate into hydrogels, their antimicrobial resistance can be improved through reduction in infections. In this respect, antimicrobial polymer/AgNPs and polymer/AgNO_3_ hydrogels for wound dressing applications have attracted considerable attention in recent years [126,127,181,182,183,184]. As an example, Oliveira et al. fabricated PVA/AgNO_3_ hydrogels loaded with 0.25% and 0.5% AgNO_3_ [184]. The nanocomposite hydrogels exhibited significant inhibition against both gram-positive and gram-negative bacteria due to the Ag^+^ ions released from silver nitrate (Figure 16a). Culturing mouse fibroblasts with nanocomposite hydrogels revealed good cell membrane integrity and cell viability (Figure 16b), indicating that nanocomposite hydrogels are non-toxic.

## 4. In Vitro Cell Cultivation

**Figure 17:**
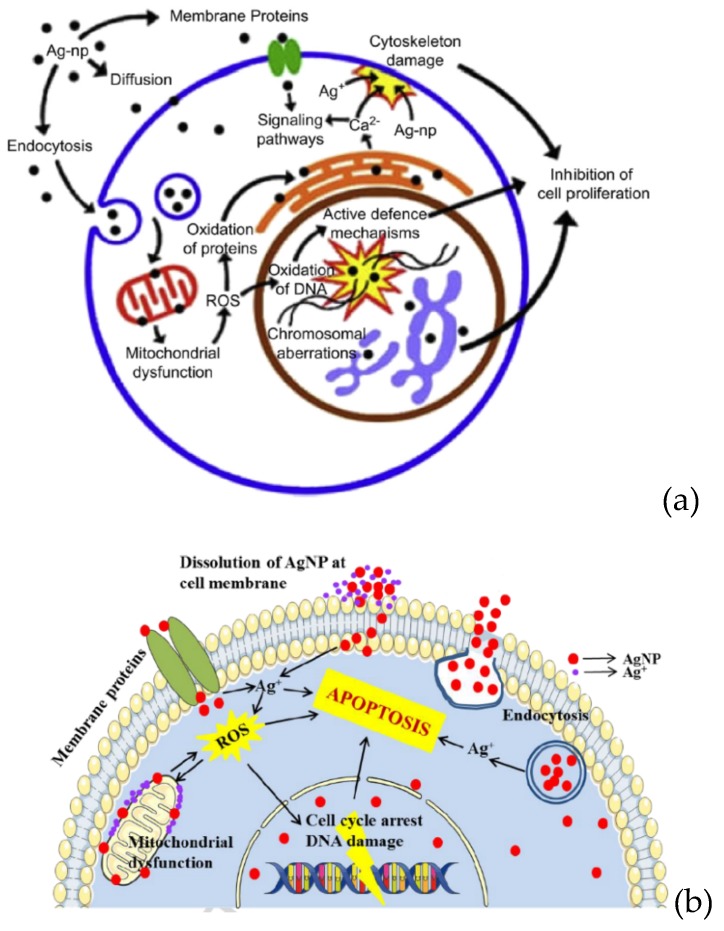
Proposed mechanisms of (**a**) AgNPs- and (**b**) silver ion-induced cytotoxicity. Reproduced from [190] and [192] with permission of BioMed Central Ltd and Elsevier, respectively.

As aforementioned, AgNPs have been widely used for antibacterial and therapeutic applications, including fabrics, food packaging materials, wound dressings, and cancer therapy [22,24,25,27,30,31,113,171,172,173,174,184]. These routes can lead to increasing exposure of AgNPs to human cells [185]. Cellular uptake of AgNPs takes place either via diffusion (translocation), endocytosis or phagocytosis [186]. Upon entering the cytoplasm, AgNPs themselves or Ag^+^ ions can generate ROS, leading to DNA damage, protein denaturation, and apoptosis [23,187]. AgNPs of different sizes and shapes tend to accumulate in the mitochondria, thereby inducing mitochondrial dysfunction, i.e., a reduction in mitochondrial membrane potential (MMP), and promoting ROS creation. This leads to the damage of intracellular proteins and nucleic acids (Figure 17a) [52,53,54,55,185,188,189,190,191,192,193,194]. Grzelak et al. and AshaRani et al. have demonstrated that the disruption of mitochondrial respiratory chain by AgNPs would increase ROS generation and interrupt ATP synthesis, thereby resulting in DNA damage [189,190]. The ROS generation can also cause cell membrane damage through the release of lactate dehydrogenase (LDH). Furthermore, AgNPs can interact with the membrane proteins and activate signaling pathways, leading to the inhibition of cell proliferation. On the other hand, Ag^+^ ions released from AgNPs can also induce ROS generation [192,195], especially for cellular uptake through endocytosis [49,51]. In this context, AgNPs confined in an acidic lysosomal environment dissolute into Ag^+^ ions. These ions initiate cascades or series of events that lead to intracellular toxicity, termed as the “lysosome-enhanced Trojan horse effect” [51]. Furthermore, some AgNPs, which translocate into cytoplasm through diffusion or channel proteins, are oxidized by cytoplasmic enzymes, thereby releasing Ag^+^ ions. Those ions interact with thiol groups of mitochondrial membrane proteins, causing mitochondrial dysfunction and generating ROS accordingly (Figure 17b). In the case of bacterial cells, several factors such as nanoparticle size, shape, surface area, surface charge, surface functionalization, and particle dispersion state also affect cytoxicity in mammalian cells [188,191]. Therefore, AgNPs tend to induce size-, dose- and time-dependent toxicity by creating ROS, oxidative stress, and DNA damage [189,193,194]. Figure 17a,b summarize possible mechanisms of AgNPs-, or Ag^+^-induced toxicity in mammalian cells [190,192].

Industrial activities involved in manufacturing AgNPs and their associated products have raised concerns over their release into the environment via several processes, including particle synthesis during manufacturing and incorporation into products, recycling, and disposal [196]. During industrial fabrication and laboratory synthesis, AgNPs in the form of powder or liquid may enter the human body through inhalation and dermal contact [197]. Inhaled nanoparticles can reach the lung alveoli, which is the deepest region of the respiratory system. AgNPs have several adverse health effects upon entering the pulmonary alveoli, because of their prolonged interaction with the lung cells. Fordbjerg et al. studied the effect of gene expression profiling in adenocarcinomic human alveolar basal epithelial cells (A549) by treating them with AgNPs [198]. They reported that AgNPs at 12.1 µg/mL modified the regulation of more than 1000 genes of A549 cells. The upregulated genes included members of the metallothionein, heat shock protein, and histone families. Moreover, ROS was also generated but did not cause apoptosis at 12.1 µg/mL AgNPs. Han et al. prepared AgNPs using both green and chemical reduction methods [193]. They demonstrated that the toxicity of AgNPs in A549 cells was dose-dependent, resulting from ROS generation and oxidative stress. In addition, green AgNPs were more toxic at lower concentrations than chem-AgNPs. The IC_50_ values for bio-AgNPs and chem-AgNPs were 25 µg/mL and 70 µg/mL, respectively. IC_50_ was the concentration of AgNPs with a 50% reduction in cell viability. Very recently, Gurunathan et al. synthesized green AgNPs from silver nitrate using biomolecule quercetin, and treated A549 with a combination of AgNPs and antitumor drug, i.e., MS-275 derived from histone deacetylases (HDACs) [199]. The as-synthesized AgNPs exhibited dose- and size-dependent toxicity against A549 cells. Combined AgNPs and MS-275 markedly induced apoptosis as a result of ROS accumulation, LDH leakage, mitochondria dysfunction, activation of caspase 9/3, up and down regulation of pro-apoptotic genes and anti-apoptotic genes, respectively. For human bronchial epithelium (BEAS-2B) cells [200,201,202], Gliga et al. employed pristine AgNPs (50 nm), PVP-AgNPs (10 nm,) and CT-AgNPs (10, 40 and 75 nm) to interact with BEAS-2B [201]. They reported that only AgNPs with a size of 10 nm induce cytotoxicity regardless of the surface coating. The cytotoxicity was associated with DNA damage and the release of intracellular Ag. Similarly, Kim et al. demonstrated that Ag-NPs induced a significant increase in the ROS level and oxidative DNA damage in the BEAS-2B cells [202].

The widespread use of antimicrobial textiles, wound dressings, and cosmetics containing AgNPs has increased human dermal exposure to those nanoparticles. Sapkota et al. demonstrated that biosynthesized AgNPs exhibit dose-dependent toxicity towards human keratinocytes (CRL-2310) [203]. At 10 µg/mL, cell viability was 98.76%, but the viability further decreased to 74.5% at 100 µg/mL. Carrola et al. indicated that Ag^+^ ions released intracellularly from CT-AgNPs caused a dose-dependent ROS generation in human skin keratinocytes (HaCaT) [49]. Further, CT-AgNPs (10 nm) agglomerated considerably in culture medium compared to CT-AgNPs (30 nm). As such, agglomerated CT-AgNPs (10 nm) became less cytotoxic than CT-AgNPs (30 nm). Avalos et al. studied genotoxic effects of PEI/PVP-coated AgNPs (4.7 nm) and uncoated AgNPs (42 nm) on normal human dermal fibroblasts (NHDFs) and human pulmonary fibroblasts (HPFs) [204]. In vitro exposure of NHDFs and HPFs to coated (0.1–1.6 µg/mL) and uncoated AgNPs (0.1–6.7 µg/mL) for 24 h triggered DNA strand fragmentation in a dose- and size-dependent manner. Furthermore, smaller PEI/PVP-AgNPs were more genotoxic than larger AgNPs. In another study, they also found that smaller AgNPs (4.7 nm) were more toxic than pristine AgNPs (42 nm) in NHDFs on the basis of MTT and LDH measurements. The oxidative stress parameters showed a dramatic increase of ROS but a depletion in glutathione levels [205].

**Figure 18:**
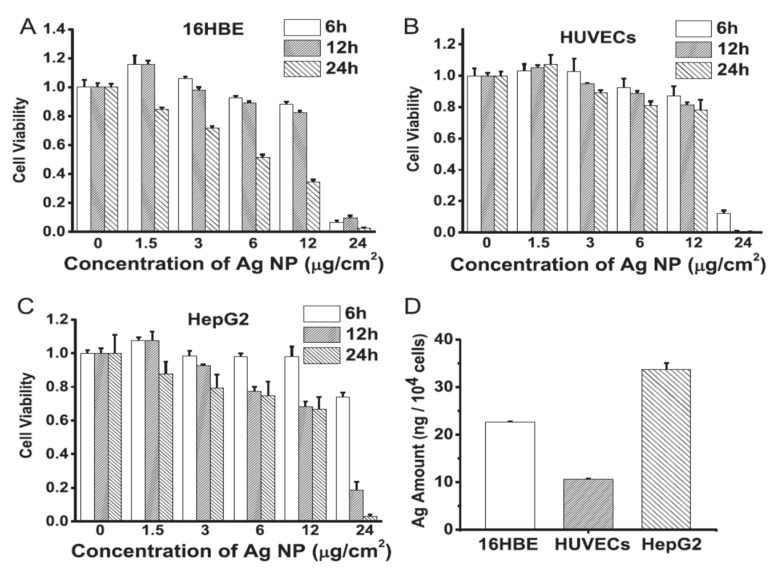
(**A**–**C**) Cell viability vs AgNP concentration for 16HBE, HUVECs and HepG2 cells determined from CCK-8 assay at different time points. (**D**) Inductively coupled plasma mass spectrometry results showing cellular uptake of AgNPs upon exposure at a dose of 2 mg/cm^2^ AgNPs for 24 h. Data are expressed as means ± SD, *n* = 5. Reproduced from [206] with permission of Elsevier.

Hou et al. studied toxicity of AgNPs (20 nm) in three human cell lines, i.e., human bronchial epithelial cells (16HBE), human umbilical vein endothelial cells (HUVECs), and human hepatocellular liver carcinoma cells (HepG2) (Figure 18A–D) [206]. HUVECs are commonly used in vitro model for assessing toxicity of nanoparticles to endothelium [207,208,209]. 16HBE cells originate from human airway epithelial cells, thus representing potential toxicity due to inhalation, while HUVECs and HepG2 cells are the target cells for AgNPs upon entering blood circulation. Human blood vessels are composed of a thin layer of endothelial cells known as the endothelium. Capillary endothelium differs in structure depending upon the tissue type in which it is located. Continuous endothelium is closely packed together and linked with tight junctions, anchored to a basement membrane. It is found in the blood vessel, skin, lung, and nervous tissues. Fenestrated endothelium is found in the capillaries of kidney and endocrine glands, while discontinuous endothelium is found in the liver. As recognized, nanoparticles come into first contact with vascular endothelium once they enter the circulation system. Vascular endothelium in different tissues has its own distinctive properties including surface receptors and intercellular junctions [209]. From Figure 18A–C, a dose- and time-dependent manner of cell viability can be readily seen, especially for 16HBE. Thus, the toxicity of AgNPs on these cell lines takes the order: 16HBE > HepG2 > HUVECs. The toxicity of 16HBE arises from the activation of endoplasmic reticulum (ER) stress signaling pathway. ER stress response is markedly induced in the 16HBE cells, but not in HUVECs and HepG2 cells [206]. Shi et al. also reported a dose-dependent toxicity of AgNPs on HUVECS [54]. In their study, AgNPs induce intracellular ROS formation, reduce cell proliferation, and cause cell membrane damage, leading to cell dysfunction and eventual apoptosis. These adverse effects are attributed to the activation of IKK/NF-κB pathways as a result of the oxidative stress. Guo et al. investigated the cytotoxicity of citrate-coated AgNPs (10, 75, and 110 nm) towards HUVECs [210]. AgNPs can be readily taken up by vascular endothelial cells, resulting in cell leakiness via altering inter-endothelial junctions.

**Figure 19:**
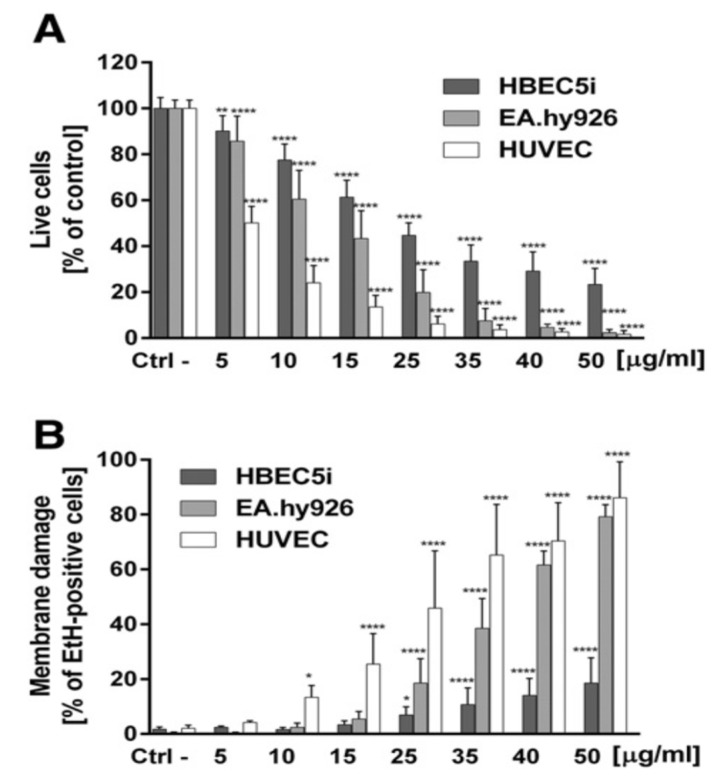
(**A**) Cell viability and (**B**) membrane damage of HBEC5i, HUVEC and EA.hy926 cells vs AgNPs concentrations after 24 h exposure to nanoparticles. Data are presented as means ± SD. * *p* < 0.05; ** *p* < 0.01; **** *p* < 0.0001. Reproduced from [59] with permission of Elsevier.

From the in vitro model in the literature, AgNPs triggered pro-inflammatory cytokines in brain endothelial cells, thereby causing an increased permeability of the cell layer. Trickler et al. studied inflammatory responses of rat brain microvessel endothelial cells (rBMECs) exposed to AgNPs of different sizes (25, 40 and 80 nm) and concentrations [58]. They reported that exposure of AgNPs to BMECs induce pro-inflammatory cytokines such as interleukin IL-1β, tumor necrosis factor (TNF-α), and prostaglandin E_2_ (PGE_2_). The pro-inflammatory response followed a size- and time-dependent manner, with IL-1β preceding both TNF-α and PGE_2_ for AgNPs (25 nm). The interactions of the Ag-NPs with endothelial cells also induced cellular damage in the form of perforations in rBMEC monolayers. The secretion of pro-inflammatory cytokines together with an increase of vascular permeability of rBMECs allowed the entry of substances into the brain tissues, inducing neuronal cell death. Very recently, Sokolowska et al. studied toxic effects induced by AgNPs on three kinds of endothelial cell lines, i.e., HUVEC, human brain endothelial cell (HBEC5i) and human endothelial cell line for blood vessel (EA.hy926) [59]. The viability of these three cell lines decreased with increasing AgNPs concentration. HBEC5i cells were much less vulnerable to AgNPs induced toxicity than EA.hy926 and HUVEC cells (Figure 19A). These three cell lines also exhibited a dose-dependent membrane damage, in which HBEC5i cells were less susceptible to the damage compared to EA.hy926 and HUVEC cells (Figure 19B). They attributed the higher cell viability against AgNPs to the presence of specialized cellular components of the brain barrier.

**Figure 20:**
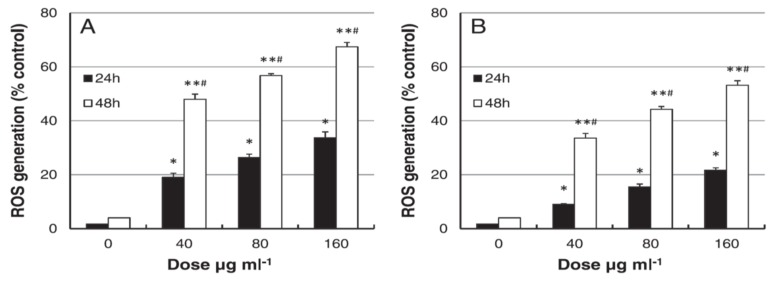
Dose-and time-dependent ROS generation in HepG2 cells exposed to AgNPs in: (**A**) deionized water and (**B**) cell culture medium. Data are expressed as means ± SD. There was significant difference between the treated and control groups (* *p* < 0.05; ** *p* < 0.01), and between the 24- and 48-h groups (# *p* < 0.05). Reproduced from [194] with permission of Wiley.

**Figure 21:**
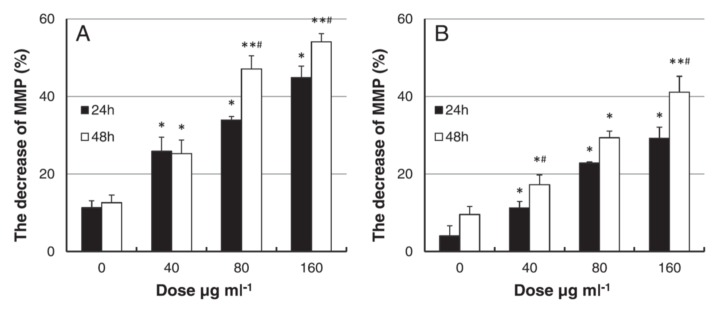
Dose-and time-dependent MMP reduction of HepG2 cells exposed to AgNPs in (**A**) deionized water and (**B**) cell culture medium. Data are expressed as means ± SD. There was significant difference between the treated and control groups (* *p* < 0.05; ** *p* < 0.01), and between the 24- and 48-h groups (^#^ *p* < 0.05). Reproduced from [194] with permission of Wiley.

The liver is one of the target organs once AgNPs enter the bloodstream [194,211,212]. Xue et al. demonstrated that AgNPs (15 nm) induce toxicity in HepG2 cells under a dose- and time-dependent manner. They also assessed the effect of solvents (deionized water and culture medium) for dispersing AgNPs on cytotoxicity [194]. The toxic effects were attributed to ROS generation, mitochondrial injury, and oxidative stress, leading to cell apoptosis (Figure 20 and Figure 21). AgNPs-induced cytotoxicity was more severe in water than culture medium because of the dissolution of AgNPs into Ag^+^ ions in water. Singh et al. biosynthesized AgNPs from silver nitrate using leaf extract of Morus alba as a reductant [212]. They then exposed green AgNPs to HepG2, and observed a dose-dependent cytotoxicity with an IC_50_ value of 20 µg/mL. The cytotoxic effect of green AgNPs was compared with the standard anticancer drug 5-Fluorouracil (5-FU) and pure Morus alba extract. The IC_50_ values of 5-FU and M. alba were recorded, respectively, as 30 and 80 µg/mL. Apparently, AgNPs showed nearly a same trend in destroying cancer cells as that of standard drug, showing potential application for hepatocellular therapy.

**Figure 22:**
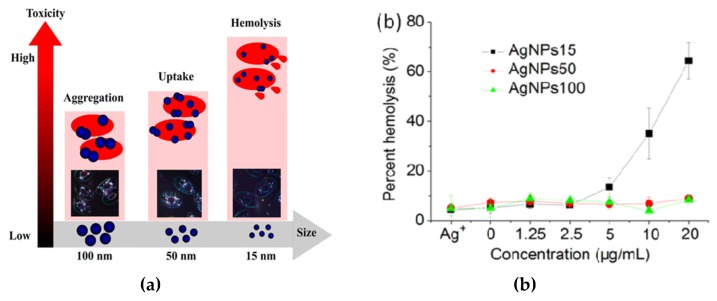

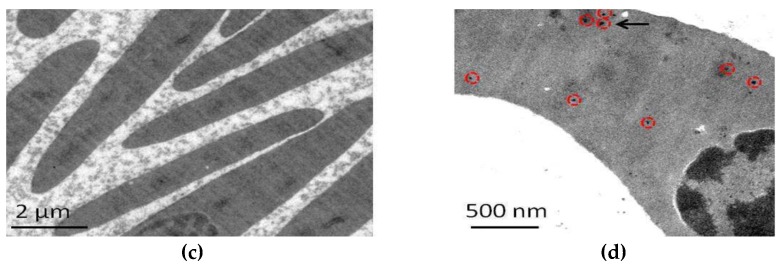
(**a**) Schematic representation showing size-dependent hemolysis of RBCs due to AgNPs. (**b**) Percentage hemolysis vs AgNPs concentrations. TEM images of RBCs (**c**) without and (**d**) with AgNPs (15 nm) treatment. Individual AgNP in (**d**) is outlined with a red circle, while AgNPs are aggregate using black arrows. Reproduced from [213] with permission of the American Chemical Society.

Red blood cells (RBCs) or erythrocytes contain no nucleus and organelles such as mitochondria; thus, they have limited repair capability following injury. Direct interaction of nanoparticles with RBCs can damage their membranes, leading to membrane rupture or hemolysis. Kim and Shin studied hemolysis, deformability, and morphological change of human RBCs exposed to AgNPs (30 and 100 nm) and silver nanowires (AgNWs) for 2 h [50]. They reported that hemolysis of RBCs is size- and dose-dependent in which small AgNPs induce higher hemolysis than large AgNPs. The shape of silver nanomaterials had little influence on hemolysis. They attributed cytotoxicity to the direct interaction of AgNPs with the RBCs, leading to the generation of oxidative stress, membrane injury, and eventual hemolysis. Chen et al. also reported a size- dependent hemolysis effect for murine RBCs (Figure 22a) [213]. Serious hemolysis was found at AgNPs (15 nm) contents ≥ 10 µg/mL (Figure 22b). Figure 22c showed the TEM image of RBCs prior to AgNPs exposure. Figure 22d showed the internalized AgNPs in RBCs, leading to membrane injury, lipid peroxidation, and eventual hemolysis. Very recently, Ferdous et al. studied the interactions of PVP and citrate coated AgNPs (10 nm) of various concentrations (2.5, 10, 40 µg/mL) with murine RBCs [214]. AgNPs induced significant dose-dependent hemolysis, resulting from cellular uptake of AgNPs and oxidative stress generation.

**Figure 23:**
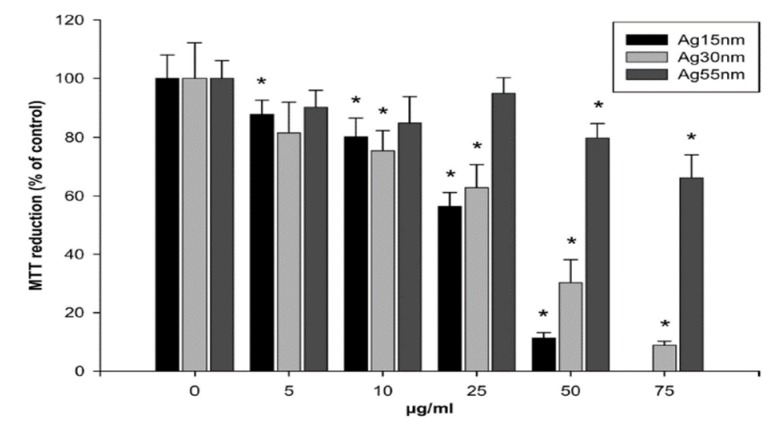
Effect of AgNPs concentration on mitochondrial metabolism (MTT assay) in murine alveolar macrophages treated with AgNPs for 24 h. The data were expressed as means ± SD (*n* = 3). *p* < 0.05 was considered significant. Reproduced from [215] with permission of the American Chemical Society.

**Figure 24:**
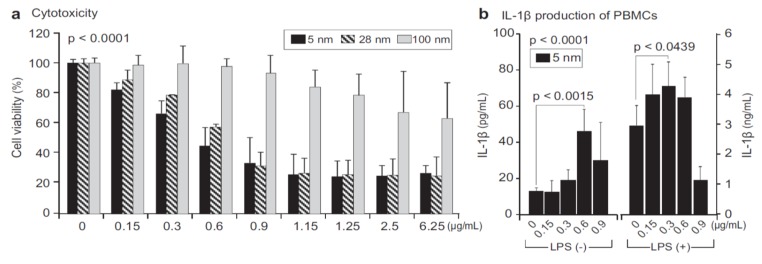
Cytotoxicity and IL-1β generation in PBMCs. (**a**) PBMCs were treated with AgNPs for 6 h and cell viability was determined with CCK-8 assay. (**b**) PBMCs were treated with AgNPs (5 nm) for 6 h and supernatant levels of IL-1β were assessed by ELISA. LPS (50 pg/mL) was pre-treated for 2 h before AgNPs exposure. Results were presented as means ± SD. One-way ANOVA analysis showed significance (*p* < 0.0001) (**a**,**b**), and Student’s *t*-test between certain pairs (**b**) was used for statistical analysis. Reproduced from [216] with permission of Elsevier.

Macrophages are well known phagocytic cells of the innate immune system, acting as a first line of defense against pathogens. They exist in nearly all mammalian tissues and are involved in bacteria killing, wound healing, restoring tissue homeostasis, and regulating immune response. AgNPs also exhibit a toxic effect on macrophages, especially those with the smallest particle sizes. Figure 23 shows typical size- and dose- dependent toxicity in murine alveolar macrophages induced by AgNPs of different sizes [215]. The IC_50_ values of AgNPs (15 nm), AgNPs (30 nm) and Ag (55 nm) were recorded as 27.87 ± 12.23, 33.38 ± 11.48, and >75 µg/mL respectively. Apparently, AgNPs (15 nm) showed the highest cytotoxicity as expected. Yang et al. studied the cytotoxic and immunological effect of AgNPs (5 nm, 28 nm and 100 nm) on innate immunity using human peripheral blood mononuclear cells (PBMCs) (Figure 24a,b) [216]. They reported a dose-dependent toxicity of AgNPs on PBMCs in which AgNPs (5 nm) were the most toxic nanoparticles. Furthermore, AgNPs with sizes of 5 nm and 28 nm induced inflammasomes to generate IL-1β and subsequent caspase-1 activation. Inflammasomes formation was derived from the leakage of cathepsins due to the disruption of lysosomal membranes, and the K^+^ efflux via cell membrane pores triggered by AgNPs. In addition, AgNPs (5 nm) and AgNPs (28 nm) increased the production of mitochondrial superoxide. At the same concentration, AgNPs (5 nm) induced more production of hydrogen peroxide that was toxic to cells [216]. Martinez-Gutierrez et al. treated the human monocytic cell line (THP-1) with AgNPs (24 nm), and reported that monocytes secrete inflammatory cytokines IL-6 and TNF-α at AgNPs contents ≥ 10 μg/mL [217]. Butler et al. examined the genotoxic effects of AgNPs (10, 20, 50 and 100 nm) on THP-1 cells, and indicated that AgNPs (10 and 20 nm) induce micronucleus nucleation and DNA strand breaks [60]. Micronucleus formation only required very low AgNPs dosages, i.e., 15 µg/mL for AgNPs (10 nm), and 20 µg/mL for AgNPs (20 nm). Silver ions released from AgNPs endocytosed by THP-1 were mainly responsible for the DNA damages.

From the literature, AgNPs can cross the brain blood barrier (BBB) through the blood circulation system [58,218]. An earlier study by Trickler et al. reported that AgNPs increased the BBB permeability in primary rat brain endothelial cells, and induced a size-dependent pro-inflammatory response by secreting PGE2, TNF-α and Il-1β [58]. Cramer et al. studied the effect of AgNPs’ surface coatings (citrate and ethylene oxide (EO) on neurotoxicity of primary porcine brain capillary endothelial cells (PBCECs) [219]. Neutral red uptake assay revealed that cell viability decreased markedly from 100% to 58% and 71%, respectively, upon exposure to EO-AgNPs and CT-AgNPs at 50 μg/mL. Furthermore, AgNPs disturbed cell barrier integrity and tight junctions, and induced oxidative stress and DNA strand breaks. Those adverse effects were reduced to a lesser extent using citrate coating. Liu et al. examined the toxic effect of AgNPs (23 nm) on embryonic neural stem cells (NSCs) from human and rat fetuses [220]. In addition, mitochondrial metabolism (MTT assay) was substantially reduced, while LDH leakage and ROS generation were markedly increased under a dose-dependent manner. AgNPs-induced neurotoxicity was further revealed by up-regulated Bax protein expression, and an increased number of TUNEL-positively stained cells [220]. From the literature, the Trojan-horse effect in murine astrocytes and microglial cells due to AgNPs uptake also led to ROS generation [189]. As such, intracellular Ag^+^ ions interacted with thiol-groups of cysteine (CYS) protein, producing Ag(CYS) and Ag(CYS)_2_ species. Yin et al. studied the effects of AgNPs (34 nm) and Ag^+^ ions in the form of silver nitrate on neurotoxicity of mouse embryonic stem cells (mESCs) [221]. They demonstrated that both AgNPs and Ag^+^ ions perturbed mESCs global and neural progenitor cell-specific differentiation processes. AgNPs and Ag^+^ ions induced anomalous expression of neural ectoderm marker genes at concentrations lower than 0.1 μg/mL [221]. Ma et al. studied the cytotoxic effect of AgNPs (30 nm) on murine hippocampal neuronal HT22 cells [222]. They reported that cytotoxicity is caused by mitochondrial membrane depolarization, increased ROS generation, and caspase-3 activation. Mitochondrial membrane depolarization results from a loss of mitochondrial membrane integrity, leading to a decrease of MMP. Caspase-3 is the main caspase responsible for apoptosis execution [223]. Apparently, brain tissue with high lipid content is particularly vulnerable to the oxidative stress. By treating HT22 cells with both AgNPs and sodium selenite, cell viability increases significantly due to selenium, and can suppress ROS generation and caspase-3 activation.

**Figure 25:**
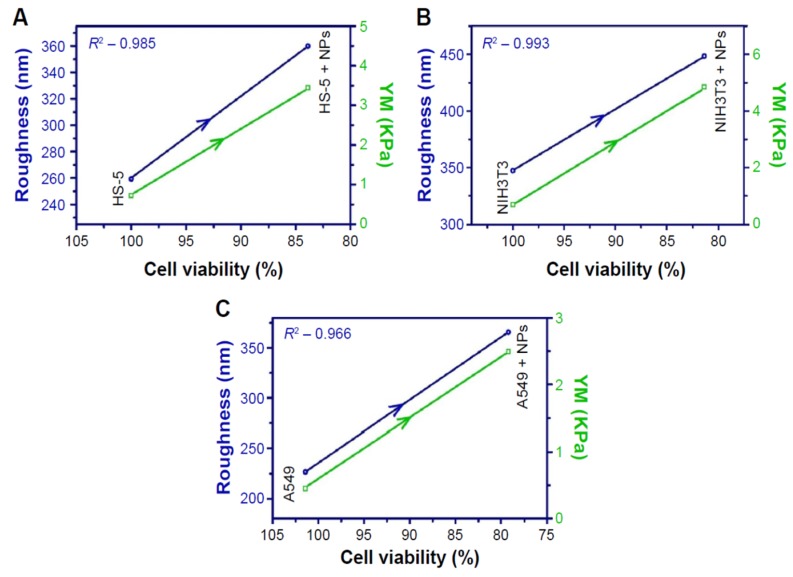
Correlation between cell viability and the roughness or stiffness of (**A**) HS-5, (**B**) NIH3T3 and (**C**) A549 cells before and after treatment with AgNPs. NP: nanoparticles; Y. M.: Young’s modulus. Reproduced from [226] with permission of Dove Medical Press Ltd.

With the fast development of material examination techniques in recent years, atomic force microscopy (AFM) has been used increasingly in the biological field [224,225]. AFM measures the surface roughness and elastic modulus of a material by moving its tip across the specimen surface. The force between the tip and the sample is measured through the deflection of cantilever during scanning [224]. For biomaterials, changes in biophysical properties (cell height and roughness) as well as biomechanics (elastic modulus) can be analyzed accordingly. Thus, AFM is a powerful tool to analyze the interaction between the cells and AgNPs at high accuracy. In this respect, Subbiah et al. employed AFM to investigate the physicomechanical responses of A549, human bone marrow stromal cells (HS-5) and mouse fibroblasts (NIH3T3) exposed to AgNPs [226]. Bioassays (CCK-8, GSH, and lipid peroxidation) were also concurrently performed. As such, the results were compared and correlated with those of AFM. From their study, AgNPs exhibited a dose-dependent reduction in glutathione (GSH), but showed an increased manner with the MDA level. AgNPs bonded directly to GSH and inhibited the enzymes for GSH synthesis, leading to GSH depletion and ROS buildup. As recognized, GSH depletion is an early event during apoptosis, which occurs before the loss of cell viability [227]. From the AFM measurements, it was seen that treatment using AgNPs leads to a substantial change in cell morphology due to enhanced cell surface roughness. Moreover, the stiffness of AgNP-treated cells also increases markedly because of the deposition of AgNPs on the cell surfaces. Figure 25A–C show the correlation between cell viability and AFM results.

More recently, Jiang et al. combined AFM and bioassays to study cytotoxic effect of AgNPs on human embryonic kidney 293T cells (HEK293T cells) [55]. In their study, AFM was used to measure cellular viscosity from the force-displacement curve. The measurements showed that cellular viscosity decreases with increasing AgNPs concentration, demonstrating that structural changes occur in kidney cells upon exposure to AgNPs. Bioassays (comet, gene expression profiling) tests showed that severe DNA damage occurs in HEK293T cells due to downregulation of antiapoptosis Bcl2-t and Bclw genes, and upregulation of the proapoptosis Bid gene. Table 2 summarizes the cytotoxic effects of AgNPs on human cell lines.

## 5. In Vivo Animal Model

An in vivo model for AgNP-induced cytotoxicity is performed directly on the tissues of a whole living animal under a controlled environment. The tests are expensive, time consuming, and subjected to several restrictions due to ethical issues. The experiments are typically performed on rodents (rats, mice and guinea pigs) through oral administration, intravenous (i.v.) injection, intraperitoneal (i.p.) injection, intratracheal (i.t.) instillation, subcotaneous injection, etc. [228]. The in vivo cytotoxic effects of AgNPs depend on several factors such as nanoparticle size and dose, administration route, exposure time and type of animal model. From published literature reports, AgNPs accumulate mainly in the target organs of animals through several administration routes, thereby inducing toxic effects such as cell dysfunction, inflammation, DNA damage, and animal death [229,230,231,232,233,234,235,236,237].

Liver is one of the main target organs for administration routes involving translocation of AgNPs in the blood circulation system [229,230,231,232,233,234,235,236,237,238,239,240]. Kupffer phagocytic cells in the liver are essential for particle removal following intravenous administration. As such, AgNPs are deposited in the Kupffer cells after injection [236,237]. Accumulated AgNPs in the liver may cause several negative effects such as the generation of ROS, pathological changes in liver morphology, and enzyme activity. Dziendzikowska et al. intravenously injected AgNPs (20 and 200 nm) to male Wistar rats at a dose of 5 mg/kg [234]. AgNPs were translocated from the bloodstream to liver, spleen, kidneys, lungs and brain, with the liver being the main target organ. Silver concentrations in these organs of the rats treated with AgNPs (20 nm) were significantly higher than those treated with AgNPs (200 nm). Furthermore, silver concentrations in these organs displayed a time- and size-dependent accumulation manner. Lee et al. intraperitoneally injected AgNPs into Sprague-Dawley (SD) rats, and reported that AgNPs accumulated mainly in the liver [235]. AgNPs caused a significant increase of caspase-3 level in the liver of treated rats from day 1 until day 30. Although autophagy was induced following i.p. injection at day 1, failure to preserve autophagy in the following days led to liver dysfunction and eventual apoptosis.

**Figure 26:**
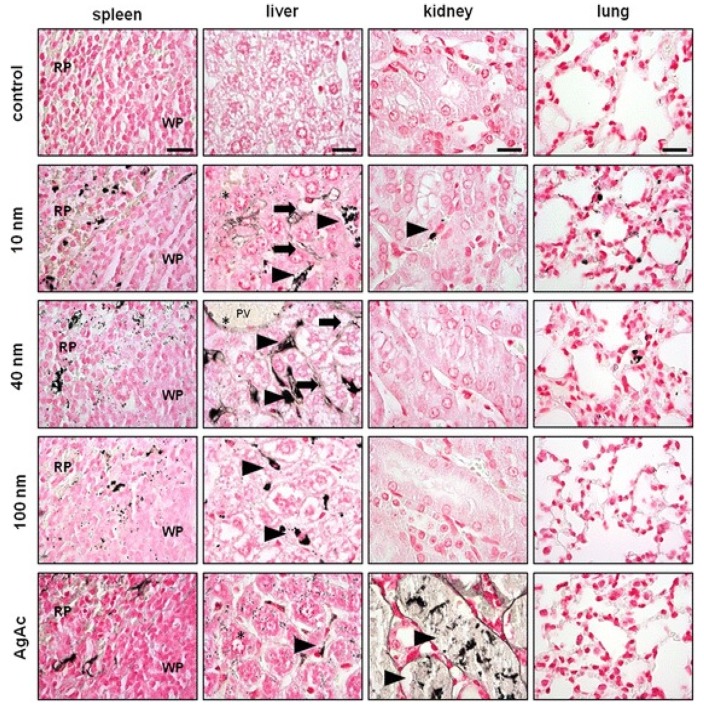
Histological examination of silver tissue localization by autometallography staining. Representative images of spleen, liver, kidney, and lung (scale bar = 20 μm), from AgNPs (10, 40, 100 nm) and silver acetate treated mice. In the spleen, silver was localized within the cytoplasm of macrophages especially in the spleen white pulp (WP) and red pulp (RP). Triangles indicate the accumulation of silver in organ tissues. Reproduced from [229], BioMed Central Ltd under the Creative Commons Attribution License.

**Figure 27:**
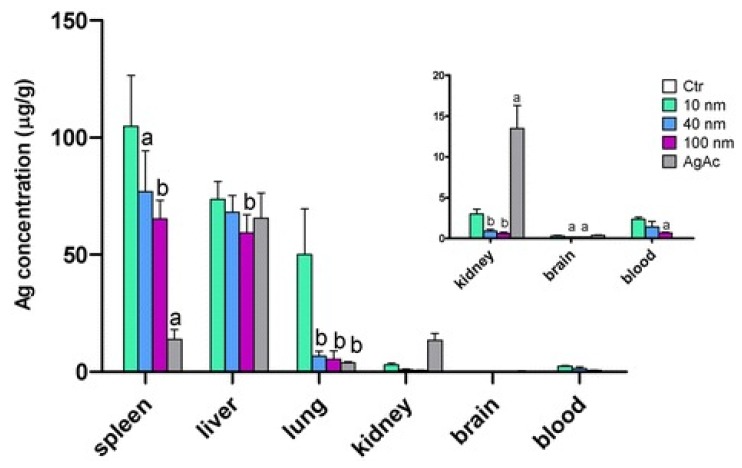
Silver tissue concentration after *i.v.* injection of AgNPs and AgAc in mice. Data are expressed as means ± SD. The inset illustrates a magnified view showing Ag concentration in the kidney, brain, and blood. Statistical significance: a = *p* < 0.05; b = *p* < 0.01. Reproduced from [229], BioMed Central Ltd under the Creative Commons Attribution License.

Recently, Recordati et al. intravenously injected CT- and PVP-coated AgNPs as well as silver acetate into CD-1 mice [229]. Commercial CT-AgNPs and PVP-AgNPs with sizes of 10, 40 and 100 nm were used in their study. Cytotoxic effects were strongly size-dependent, while coating type (CT or PVP) had no impact on biodistribution of AgNPs in the organ tissues. Histological examination revealed that AgNPs were predominantly accumulated in the spleen and liver, and to a lesser extent in the kidney and lung (Figure 26). Very high silver concentrations were detected by inductively coupled plasma mass spectrometry (ICP-MS) in the spleen and liver, followed by lung, kidney and brain. AgNPs (10 nm) were found to be the most toxic nanoparticles (Figure 27). Silver acetate (AgAc) at the same dosage (10 mg/kg) was also detected in these organs after administration. Very recently, Yang et al. also demonstrated that AgNPs (3 nm) were mainly deposited in the liver and spleen of male mice, followed by the kidney, heart, lungs and testis, and the least accumulation was found in the stomach, intestine following *i.v.* injection [230]. RT-qPCR analysis of the liver revealed substantial changes in the gene expression profiles, i.e., upregulation of several genes such as *p*53, caspase-3, caspase-8, transferrin (Trf), and Bcl-2. As is known, caspases are enzymes that cause apoptosis by cleaving cellular proteins. Initiator caspases such as caspase 2, 8, 9 and 10 initiate the apoptotic process, leading to the activation of effector caspases, i.e., caspase 3, 6 and 7 [222]. Wen et al. intravenously injected SD rats with CT-AgNPs (6.3 nm) at a dosage of 5 mg/kg body weight (bw) respectively [231]. They reported that the lungs, spleen, and liver were enriched with Ag content on the basis of ICP-MS measurements. In addition, the silver concentration distribution in the organs from highest to lowest took the following sequence: lung > spleen > liver > kidney > thymus > heart. Furthermore, AgNPs induced chromosome aberration in bone marrow cells.

From in vitro cell cultivation, AgNPs increased the permeability of tight junctions of brain endothelial cells [219]. The ICP-MS measurements of in vivo animal model showed the presence of a small amount of AgNPs in the mice brain [229,232,234]. Thus, AgNPs can cross the brain blood barrier (BBB) through the bloodstream, thereby inducing neurotoxicity and neuronal death. Hadrup et al. reported that AgNPs (14 nm) with doses of 4.5 and 9 mg/kg bw/day and ionic silver in the form of silver acetate (9 mg/kg bw/day) increased the dopamine concentration in the brain of female rats following 28 days of oral administration, resulting in cellular apoptosis [241]. Wen et al. conducted intranasal instillation of PVP-AgNPs (26.2 ± 8.9 nm) in neonatal SD rats with doses of 0.1 and 1 mg/kg bw/day, and ionic silver in the form of silver nitrate for 4 and 12 weeks, respectively [232]. Dose-dependent silver accumulation occurred for both AgNPs and silver ions in the liver, lung and brain. The highest silver concentration was found in the liver at week 4, while it shifted to the brain after week 12. Their findings revealed the potential neuronal damage from the intranasal administration of AgNPs or silver colloid-based products [232]. Xu et al. administered intragastrically a low dose (1 mg/kg bw/day) and a high-dose (10 mg/kg bw/day) into SD rats for 14 days [242,243]. A low dosage induced neuron shrinkage and astrocyte swelling. The adverse effect of AgNPs was attributed to the presence of lymphocytes around astrocytes. More recently, Dabrowska-Bouta et al. investigated the influence of AgNPs on the toxicity of cerebral myelin [244]. In that study, Wistar rats were exposed to 0.2 mg/kg bw per day of AgNPs (10 nm) via the gastrointestinal route. They observed enhanced lipid peroxidation and decreased concentrations of protein and non-protein –SH groups in myelin membranes.

**Figure 28:**
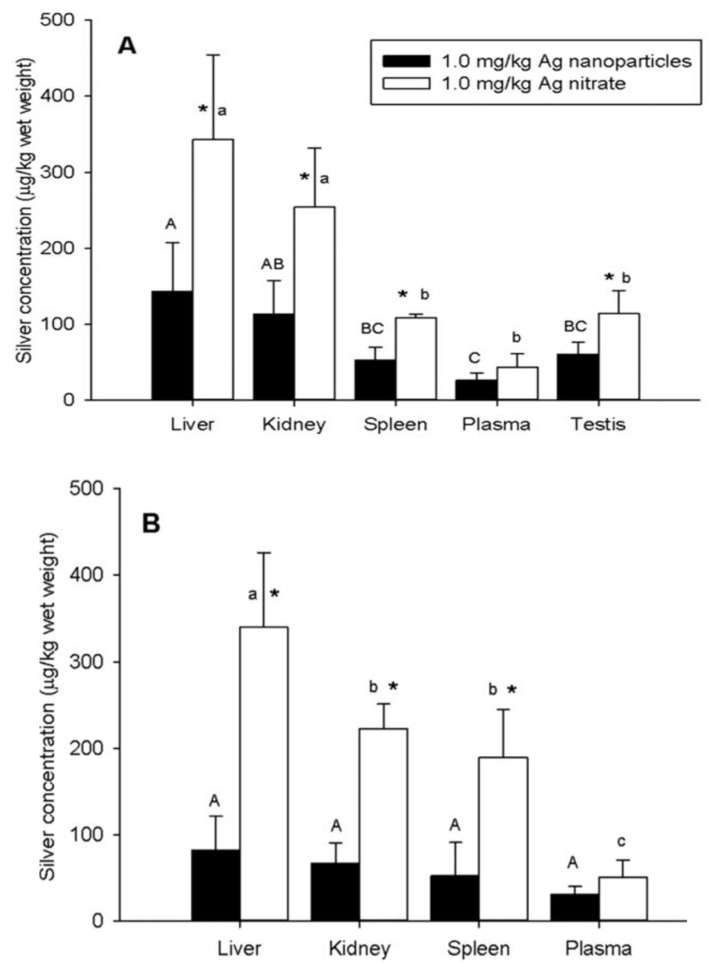
Silver concentrations in major organs and plasma of (**A**) male and (**B**) female rats. Values are presented as means ± SD, *n* = 5. The asterisk (*) indicates significant difference between AgNPs and AgNO_3_ treatment groups at *p* < 0.05. Means with the same capital letters are not significantly different among AgNPs groups (*p* < 0.05) and same small letter are not statistically different among AgNO_3_ groups by the Tukey test (*p* < 0.05). Reproduced from [238] with permission of Wiley.

We now consider cytotoxic effects induced by AgNPs and ionic silver in mice following oral administration [238,245,246,247,248]. Liver and kidney are the main target organs for mice administered orally with AgNPs. These organs play crucial roles in the clearance of exogenous substances. Bergin et al. administered CT- and PVP-AgNPs with sizes of 20 and 110 nm, and doses of 0.1, 1 and 10 mg/kg into Black-6 mice for three days through oral gavage [245]. Nearly 70.5–98.6% of administered AgNPs was excreted in feces following oral administration. Thus, no toxicity and significant tissue accumulation of AgNPs were found in treated mice. Boudreau et al. introduced AgNPs of different sizes and dosages into SD rats via oral gavage [246]. They found low accumulation of silver in tissues of rats treated with AgNPs of larger sizes, i.e., 75 and 100 nm. In contrast, tissues from rats treated with smaller AgNPs (10 nm) at 36 mg/kg bw/day showed significant silver accumulation in the kidneys, spleen and liver. In the kidneys, silver was localized within the renal tubular epithelium. Qin et al. studied the toxicity of PVP-AgNPs and AgNO_3_ in male and female SD rats treated with repeated oral administration at doses of 0.5 and 1 mg/kg bw daily for 28 days. They found no significant toxic effects of AgNPs and AgNO_3_ up to 1 mg/kg in terms of the body weight, organ weight, food intake, and histopathological examination [238]. However, ICP-MS results revealed the presence of silver in the liver, kidney, spleen and, plasma (Figure 28A,B). The total Ag contents in organs were significantly lower in the AgNPs-treated rats than those in the AgNO_3_ treated rats. In addition, silver was detected in the testis of male rats. Statistical difference in silver concentrations was found in major organs of male rats treated with AgNPs, while no difference of Ag distributions was observed in female rats. The gender-related difference in AgNPs’ distribution may be related to hormonal regulation in these organs. van der Zande et al. also indicated that silver contents in the liver, spleen, testis, and kidney of rats are mainly derived from the Ag^+^ ions of AgNO_3_, and to a much lesser extent from AgNPs after oral administration for 28 days [248].

Environmental airborne AgNP levels (5–289 mg/m^3^) in occupational settings such as factories or laboratories are harmful to the lung tissues of humans due to the inhalation of nanoparticles [249]. Therefore, intratracheal instillation and inhalation in animal models provide relevant information for assessing toxicity arising from airborne nanoparticles [250]. Studies on intratracheally instilled AgNPs into mice have been carried out in recent years [57,251,252,253,254]. For instance, Anderson et al. studied the effects of size, surface coating, and dose on the persistence of silver in the lung of the rats through *i.t.* instillation of AgNPs for 1, 7, and 21 days. Silver retention in the lung was assessed at those mentioned timepoints. Four different AgNPs: 20 nm or 110 nm in size and coated with either citrate or PVP, at 0.5 mg/kg and 1.0 mg/kg doses were adopted in their study [251]. These dosages were chosen to simulate an environmental particle exposure (5–289 mg/m^3^) in manufacturing industries [249]. CT-AgNPs was found to persist in the lung up to 21 days with retention higher than 90%, while PVP-AgNP showed lower retention in the lung, i.e., <30%. As a result, CT-AgNPs triggered lung macrophages for nanoparticle clearance. Larger nanoparticles were more rapidly cleared from the lung airways than smaller particles. Table 3 lists the recent in vivo animal studies relating cytoxicity of AgNPs through different administration routes.

In this review, we have discussed many cases relating the toxic effects of AgNPs in mammalian cells under in vitro and in vivo conditions. However, AgNPs would show no cytotoxicity toward mammalian cells and have high antibacterial efficacy in certain cases. AgNPs capped with appropriate polymers at certain concentrations do not exhibit cytotoxicity. Jena et al. reported that chitosan-capped AgNPs exhibit antibacterial activity against *P. aeruginosa*, *S. typhi*, and *S. aureus*, and they do not exhibit cytotoxic effects on mouse macrophage cell line (RAW264.7) at the bactericidal concentration [255]. Tam and coworkers demonstrated that CT-AgNPs promoted wound healing in mice through the modulation of fibrogenic cytokines, in addition to their antimicrobial properties [21,28]. Furthermore, AgNPs enhanced the differentiation of fibroblasts into myofibroblasts, thereby promoting wound contraction [28]. Pallavicini et al. synthesized AgNPs and coated with a biopolymer peptin acting both as a reductant and a stabilizing agent [29]. The as-synthesized AgNPs showed bactericidal activity against *E. coli* and *S. epidermidis*, and also facilitated normal human dermal fibroblasts (NHDFs) proliferation and wound healing on model cultures. Alarcon et al. functionalized AgNPs with thiol-LL37 cathelicidin peptide (LL37-SH), and then incorporated them into collagen hydrogels [26]. In in vitro experiments, the resulting hydrogel nanocomposites exhibited high antibacterial activity against *P. aeruginosa*, while showing no toxicity toward HUVEC and human corneal epithelial cell (HCEC). Finally, subcutaneous implantation of hydrogel nanocomposites into mice did not increase the secretion of pro-inflammatory cytokine IL-6 [26].

## 6. Conclusions

The article provides a comprehensive and state-of-the art review on the synthesis of AgNPs, their antibacterial activity, and cytotoxic effect in mammalian cells. The bactericidal activity of AgNPs has led to their widespread use in cosmetics, medical products, antimicrobial dressings, etc. However, the extensive use of AgNPs has raised significant public concerns over the safety and environment impacts of these products. In this respect, it deems necessary to study the interaction between AgNPs and biological cells in order to achieve a better understanding of the health risks arising from the use of nanoparticles. AgNPs have been shown to be toxic to numerous bacterial strains. The antibacterial activity against both gram-negative and gram-positive bacteria is found to be size-, shape-, dose-, charge- and time-dependent. Several studies have revealed that the membrane damage, mitochondrial dysfunction, ROS generation, oxidative stress and DNA damage are responsible for the cellular damage of treated bacterial cells. However, the exact bactericidal mechanisms of AgNPs remain unclear. The bactericidal effect may arise from either AgNPs themselves, released silver ions, or a combination of both.

Cell culture studies revealed that AgNPs are able to induce cytotoxicity in human cell lines including human bronchial epithelial cells, HUVECs, red blood cells, macrophages, liver cells, etc., particularly for those with sizes ≤10 nm. The cytotoxicity of AgNPs has been reported to be a dose-, size- and time-dependent manner. Similarly, there is much debate in the literature on whether AgNPs or silver ions exert toxic effects in mammalian cells. In vivo animal model tests have shown that AgNPs can pass the BBB of mice through the circulation system, thereby inducing neurotoxicity and neuronal death. Furthermore, AgNPs tend to accumulate in mice organs such as liver, spleen, kidney and brain following intravenous, intraperitoneal, and intratracheal routes of administration.

In spite of the widespred use of AgNPs in healthcare and cosmetic applications, several challenges remain to be overcome. The development of AgNPs and their nanocomposites, having both antimicrobial properties and no cytotoxic effects, is crucial for treating bacterial infections. AgNPs have been shown to be nontoxic in mouse fibroblasts, NHDFs and HCECs [21,26,28,29]. However, they are considered to be toxic to most human cell lines. More recently, biosynthesized AgNPs have been reported to be effective in killing multidrug-resistant bacteria [38,40,106,107]. Green synthesis of AgNPs generally suffers from some drawbacks, such as selection of appropriate plant extract, long reaction time, and difficulty in controlling the size and shape of AgNPs. The nature of biomolecules present in the plant extracts plays a crucial factor in the biosynthesis of AgNPs. In this respect, the quality of selected extract is considered to be of great importance [256]. Thus, it needs a systematic, reproducible, and scaled-up process for preparing green AgNPs with desirable antimicrobial properties and low toxicity. Finally, many in vitro studies in the literature employed several specific bioassays for evaluating cytotoxic effects of AgNPs. Standardization of bioassays is useful because it can provide a reliable and reproducible data for evaluating the mechanism responsible for cytotoxicity of AgNPs.

## Figures and Tables

**Table 1:** Bacterial reduction percentages of PVP-AgNP/nylon nanocomposite fabrics against *E. coli* and *S. aureus*. Reproduced from [134] with permission of Elsevier.

Bacterium	AgNP Content (ppm) in Fabrics	Percentage of Bacterial Reduction		Number of Washing	
		0	10	20	30
*E. coli*	100	99.99	99.99	99.46	99.20
*E. coli*	200	99.99	99.99	99.99	99.55
*S. aureus*	100	99.99	99.86	99.27	86.92
*S. aureus*	200	99.99	99.57	99.27	91.03

**Table 2:** Cytotoxic effects of AgNPs on human cell lines.

Synthetic Route and Size	AgNPs Dosage and Exposure Time	Cell Type	Cytotoxic Effect	Ref.
Green & chemical reduction; 15 nm	10, 20, 30, 40 and 50 µg/mL for 24 h	A549	ROS creation, MMP reduction, LDH leakage, phagocytosis	[193]
Green synthesis; 11 nm	AgNP (1 µM) + MS-275 (1 µM) for 24 h	A549	Apoptosis due to ROS creation, LDH leakage, mitochondria dysfunction, DNA fragmentation	[199]
Chemical reduction; 15.9 ± 7.6 nm	12.1 µg/mL for 24 and 48 h	A549	Exposure of AgNPs for 24 h altered the regulation of more than 1000 genes; ROS generation	[198]
Chemical reduction; 19.5 nm	1.25, 2.5, 5, 10, 20 and 40 µg/mL for 24 h	A549, HS-5; NIH3T3	AgNPs treatment increased surface roughness and stiffness of the cells.	[226]
Commercial particles; CT-AgNPs: 10, 40, 75 nm; PVP-AgNPs: 10 nm	5, 10, 20 and 50 µg/mL for 24 h	BEAS-2B	Size-dependent toxicity. AgNPs with 10 nm were more toxic, leading to DNA damage without ROS generation	[201]
Commercial particles; CT-AgNPs: 10, 30 and 60 nm	10 and 40 µg/mL for 24 h and 48 h	HaCaT	Dose-dependent ROS generation	[49]
Green synthesis; 20 nm	10, 20, 40, 60, 80 and 100 µg/mL for 24 h	CRL-2310	Dose-dependent toxicity. Cell viability was 98.76% at 10 µg/mL, but reduced to 74.5% at 100 µg/mL	[203]
Commercial particles; Pristine AgNPs: 42 nm; PEI/PVP coated-AgNPs: 4.7 nm	AgNPs: 0.1, 0.5, 1.6 and 6.7 µg/mL. Coated AgNPs: 0.1, 0.5, 0.8, 1.6 µg/mL	HPF and NDHF	DNA strand breaks in a dose- and time-dependent manner. Smaller coated-AgNPs were more genotoxic than larger pristine AgNPs	[204]
Chemical reduction; 65 nm	0.5, 1, 1.5 and 2 µg/mL	HUVEC	Dose-dependent toxicity. ROS creation and cell dysfunction via IKK/NF-κB pathways	[54]
Commercial particles; <100 nm	5, 10, 15, 25, 35, 40 and 50 µg/mL for 24 h	HBEC5i; HUVEC; EA.hy926	Cell viability and membrane damage were dose-dependent.	[59]
Commercial particles; 15 nm	40, 80 and 160 µg/mL for 24 h and 48 h	HepG2	Dose-dependent cytotoxicity. ROS creation, MPP reduction & apoptosis	[194]
Green synthesis; 10–50 nm	1, 5, 10, 20, 40 and 80 µg/mL for 24 h	HepG2	Dose-dependent cytotoxicity; IC_50_ = 20 µg/mL	[212]
Commercial particles; 60 nm	10, 20 and 40 µg/mL for 24 h	HEK293T	Decreased cell viability, increased DNA damage by exposing to AgNPs with increasing concentration	[55]
Chemical reduction; AgNPs: 30 and 100 nm AgNWs: length (1–2 µm)	100, 200, 300, 400 and 500 µg/mL for 2 h	Human erythrocyte	Size- and dose- dependent hemolysis	[50]
Commercial particles; 5, 28 and 100 nm	0.15, 3, 6, 9, 1.15, 1.25, 2.5 and 6.25 µg/mL for 6 h	PBMC	Dose-dependent cytotoxicity. AgNPs induced inflammasomes to produce IL-1β.	[216]
Green synthesis; 24.4 nm	2, 5, 6.25, 10, 12.5, 50 µg/mL for 24 h	THP-1	Cell death more than 42% at 12.5 µg/mL AgNPs. Induced cytokines IL-6 and TNF-α	[217]
Commercial particles; 10, 20, 50 and 100 nm	1, 2.5, 5, 10, 15 and 25 µg/mL for 24 h	THP-1	AgNPs (10 nm) and AgNPs (20 nm) induced DNA damage	[60]
Chemical reduction; 23 nm	1, 5, 10, 20 µg/mL for 24 h	NSC	Reduction in mitochondrial metabolism; increased LDH leakage and ROS level	[220]

**Table 3:** Recent in vivo studies showing biodistribution and toxicity of AgNPs in rodents through different administration routes.

Coating Type & Size of AgNPs	Model	Dosage & Exposure Time	Entry Route	Cytotoxic Effect	Ref.
CT and PVP; 10, 40 &100 nm	CD-1 Mice	10 mg/kg bw;24 h	*i.v.*	Biodistributed in spleen and liver followed by lung, kidney and brain. AgNPs (10 nm) are the most toxic nanoparticles	[229]
Carboxyl; 3 nm	KM Mice	11.3–13.3 mg/kg bw; 4 weeks	*i.v.*	Biodistributed mainly in spleen and liver, followed by kidney, lung, heart and testis	[230]
CT; 6.3 nm	SD rats	5 mg/kg bw; 24 h	*i.v.*	Biodistributed in the organs with decreasing Ag concentration, i.e., lung > spleen > liver > kidney > thymus > heart	[231]
PVP; 26.2 nm	SD rats	0.1 and 1 mg/kg bw per day for 4 and 12 weeks	Intranasal instillation	Dose- and time-dependent accumulation of both AgNPs and silver ion (AgNO_3_) in liver, lung and brain	[232]
2 and 20 nm	Wistar rats	5 mg/kg bw; 1, 7and 28 days	*i.v.*	Time- and size-dependent accumulation of AgNPs in the liver, spleen, kidney and brain	[234]
PVP; 10–30 nm	SD rats	500 mg/kg bw; 1, 4, 7, 10 and 30 days	*i.p.*	AgNPs located mainly in the liver. A significant increase in caspase-3 in the liver of treated rats from day 1 to day 30	[235]
CT; 3–10 nm	SD rats	1mg/kg bw. and 10 mg/kg bw for 14 days	Intragastric	Neuron shrinkage, cytoplasmic or foot swelling of astrocytes and inflammation	[229]
CT; 10 nm	Wistar rats	0.2 mg/kg bw per day for 14 days	Gastro-intestinal	Enhanced lipid peroxidation and decreased concentrations of protein and non-protein –SH groups in myelin	[243]
CT, PVP; 20 and 110 nm	Black 6 mice	0.1, 1, 100 mg/kg bw per day for 3 days	Oral gavage	No toxicity and no significant tissue accumulation	[245]
CT; 10, 75 & 110 nm	SD rats	9, 18, 36 mg/kg bw for 13 weeks	Oral gavage	AgNPs predominantly deposited within cells of major organs	[246]
PVP; 28–43 nm	SD rats	0.5 and 1 mg/kg bw daily for 28 days	Oral admi-nistration	Biodistributed in liver, kidney, spleen and blood plasma.	[238]
CT, PVP; 20 and 110 nm	SD rats	0.5 and 1 mg/kg bw for 1, 7 and 21 days	*i.t.* instillation	CT-AgNPs persisted in the lung to 21 days with retention >90%, while PVP-AgNP had lower retention of less than 30%. CT-AgNPs triggered lung macrophages for clearance of AgNPs	[251]
